# *Eif2s3y* Promotes the Proliferation of Spermatogonial Stem Cells by Activating ERK Signaling

**DOI:** 10.1155/2021/6668658

**Published:** 2021-01-29

**Authors:** Mengfei Zhang, Na Li, Wenqing Liu, Xiaomin Du, Yudong Wei, Donghui Yang, Zhe Zhou, Fanglin Ma, Sha Peng, Shiqiang Zhang, Xin He, Chunling Bai, Guangpeng Li, Jinlian Hua

**Affiliations:** ^1^College of Veterinary Medicine, Shaanxi Centre of Stem Cells Engineering & Technology, Northwest A&F University, Yangling, Shaanxi 712100, China; ^2^State Key Laboratory of Reproductive Regulation and Breeding of Grassland Livestock, School of Life Sciences, Inner Mongolia University, Hohhot 010070, China

## Abstract

The future fertility of males with cancer may be irreversibly compromised by chemotherapy and/or radiotherapy. Spermatogonial stem cell transplantation is believed to be a way to restore fertility in men. However, the survival efficiency of transplanted cells is still low. Eukaryotic translation initiation factor 2 subunit 3 and structural gene Y-linked (*Eif2s3y*) located on the Y chromosome of male animals is a coding gene of eIF2*γ* which mainly functions in translation initiation. Recently, the emerging role of *Eif2s3y* in spermatogenesis has been emphasized in several studies. However, the underlying mechanism is still unclear. In addition, how *Eif2s3y* functions in large animals remains largely unknown. In this study, we obtained the CDS sequence of the *Eif2s3y* gene from the testis of dairy goats and found that this gene was highly expressed in the testis and was evolutionarily conserved among different species. Interestingly, overexpression of *Eif2s3y* promoted the proliferation of spermatogonial stem cells of dairy goats by activating the ERK signaling pathway. In animal experiments, overexpressing *Eif2s3y* promoted transplanted goat spermatogonial stem cells and produced more colonies after microinjection into the seminiferous tubules of infertile mice. In conclusion, our study highlights an undiscovered role of *Eif2s3y* in dairy goat reproduction. This finding may provide an important basis for future works regarding male spermatogenic cell restoration and represent a major advance toward surrogate sires becoming a tool for disseminating and regenerating germplasm in all mammals.

## 1. Introduction

Spermatogenesis is essential for the continuation of most species. The reduction of spermatogonial stem cells (SSCs) can destroy spermatogenesis and leads to male infertility [1, 2]. In addition to maintaining stable spermatogenesis, studies in mice have shown that a small fraction of undifferentiated spermatogonia can regenerate spermatogenic lineage after being isolated from donor tissues and transplanted into the testis of recipient males lacking endogenous reproductive lines [3]. These regenerated spermatogonia are often referred to as spermatogonial stem cells. SSCs are located on the basement membrane of seminiferous tubules, and the delicate control of SSC self-renewal and differentiation critically determines sperm production in male animals [2, 4]. Therefore, a defect in SSC proliferation usually results in reduced germ cell number or even male infertility [5].

Chemotherapeutic drugs, such as busulfan and cisplatin, cause male reproductive damage and long-term infertility by damaging SSCs [6, 7]. In human reproductive medicine, SSCs can be used to solve infertility caused by spermatogenesis and maturation disorders [8]. Spermatogonial stem cell transplantation (SSCT) has many potential applications and may have a significant impact on society. Successful spermatogenesis has not been achieved following the transplantation of human testis tissue. However, there have been successful cases of animal SSCT, such as mice, dogs, and nonhuman primates [9, 10]. Thus, improving the proliferation ability of SSCs is critical for the rapid restoration of male reproductive capacity.

Eukaryotic translation initiation factor 2 subunit 3 and structural gene Y-linked (*Eif2s3y*) is located on the Y chromosome of male animals and is traditionally considered to be involved in the formation of the eIF2 polymer to mediate translation initiation [11, 12]. In recent years, several studies have shown that *Eif2s3y* is essential for mouse spermatogenesis [13, 14]. In 2014, Yamauchi et al. reported that mouse progeny could be generated by male germ cells with the Y chromosome contribution limited to only two genes, *Sry* and *Eif2s3y* [15, 16]. Importantly, *Eif2s3y* may be the only Y chromosome gene required to drive mouse spermatogenesis. In our previous studies, increased efficiency of haploid cell induction has been detected in *Eif2s3y*-overexpressing (*oeEif2s3y*) embryonic stem cells (ESCs) [17]. However, how *Eif2s3y* improves the efficiency of spermatogenesis is still unclear.

In the present study, we wanted to explore the role and regulatory mechanism of *Eif2s3y* in dairy goats. We obtained the *Eif2s3y* gene fragment of dairy goats and found that the expression level of *Eif2s3y* in the testis was significantly higher than that in other tissues. In addition, we found that *Eif2s3y* promoted goat SSC proliferation dependent on the extracellular regulated protein kinases (ERK) signaling pathway. The SSCT experiment showed that *Eif2s3y* could increase the number of SSCs transplanted into busulfan-treated mice. Our study may provide an efficient approach for the repair of male spermatogenic cells in large animals and improve the efficiency of livestock genetic breeding in the future.

## 2. Materials and Methods

### 2.1. Animal Experiments

All animal experiments were performed in accordance with the Guide for the Care and Use of Laboratory Animals (Ministry of Science and Technology of the People's Republic of China, Policy No. 2006 398) and were approved by the Animal Care and Use Center of the Northwest A&F University.

Different tissues and testes at different ages (1, 3, 6, 9, 12, 18, and 24 months) of Guanzhong dairy goats were supplied by Yaoan slaughterhouse in the Yangling Agricultural High-tech Industrial Demonstration Zone. Three male goats from each age were used in the testis collection. These tissues were then used to extract RNA by using RNAiso Plus (#9109, Takara Bio Inc., Japan).

The male ICR mice used for the infertile mouse model were purchased from Dashuo Laboratory Animal Limited Company in Chengdu, China. Twenty 7-week-old male mice were treated with busulfan (B2635-25G, Sigma-Aldrich by Merck) at a dose of 30 mg/kg for 2 weeks to be rendered infertile. These busulfan-treated mice were used for spermatogonial transplantation [1, 18].

### 2.2. Cell Culture and Preparation of Dairy Goat SSCs

The procedures for isolating and purifying SSCs were in accordance with a previous study, and the morphology and function of SSCs we used have been verified [19–21]. The procedures for isolating and purifying SSCs are as follows. Testes from dairy goats of 3 months were aseptically collected. After washing five times with phosphate-buffered saline (PBS) containing 100 U/mL penicillin and 100 mg/mL streptomycin, testes were cut into small pieces by using sterile scissors. Seminiferous epithelial cells were incubated with an enzyme cocktail containing 0.1% collagenase IV (Invitrogen) and 10 *μ*g/mL DNase I (Sigma-Aldrich by Merck) at 37°C for 30 min, and the cell suspension was blended every 10 min at the same time. The dissociated fragments were then digested with 0.25% trypsin (Invitrogen) for 15 min, followed by neutralization with Dulbecco's modified Eagle's medium (DMEM, Invitrogen, Carlsbad, CA, USA) containing 10% FBS (Gibco, MA, USA). The cell suspension was then filtered by 40 *μ*m copper meshes to exclude the seminiferous tubules. Then, the cell suspension was plated in culture dishes and incubated in an atmosphere composed of 5% CO_2_ at 37°C for 2 hours.

Nonadherent SSCs were obtained and removed to a new dish when the Leydig cells attached to the culture dish. Then, these cells were purified by the MASC technique to obtain Thy1-positive cells. Dairy goat SSCs were cultured in a medium containing DMEM/F12 (Invitrogen) with 1% FBS, 10% KSR (Invitrogen), 0.1 mM *β*-mercaptoethanol (Sigma-Aldrich by Merck), 1% nonessential amino acids (Invitrogen), 1% L-glutamine (Invitrogen), 10 ng/mL basic fibroblast growth factor (bFGF, Millipore), 10 ng/mL GDNF (Reproach), 50 ng/mL Gfra1 (Sino Biological, Inc., Beijing, China), and 20 ng/mL epidermal growth factor (EGF, Sino Biological, Inc.) [22, 23]. These cells were cultured for 12 hours at 37°C, supplemented with 5% CO_2_ in the air. The medium was refreshed every day. The dairy goat SSCs were passaged by TrypLE (Invitrogen).

### 2.3. Seminiferous Tubule Transplantation

For SSCT, approximately 100 *μ*L of a single cell suspension or medium was injected through the efferent duct into the left testis or right testis of busulfan-treated mice, respectively. The testis which was injected with the medium was the control group. The seminiferous tubule injection protocol was conducted as previously reported [24, 25]. These testes were collected for analysis 4 weeks after injection [26].

### 2.4. Construction of Recombination Plasmid

The primer sequences for the dairy goat *Eif2s3y* CDS clone which were designed according to the published *Mus musculus Eif2s3y* mRNA sequence (XM_006531609) were as follows: forward: 5′-AGAATTCTTCGGCAAGATGGCG-3′, reverse: 5′-AGCGGCCGCCTTCATTCATCATC-3′.

*Eif2s3y* was amplified from the dairy goat testicular cDNA by a reverse transcription-polymerase chain reaction. Then, the specific fragments were cloned into the pCDH-CMV-MCS-EF1 vector. Nucleotide fragments for knocking down experiments which were sent to biological companies for synthesis were as follows: 5′-CCGGGAACAGATACTTGCATTTGTACTCGAGTACAAATGCAAGTATCTGTTCTTTTTTG-3′. The specific nucleotide fragments were cloned into the CD513B-U6-*shEif2s3y* vector. The recombinant plasmid pCDH-CMV-*Eif2s3y*-EF1-puro (*oeEif2s3y*), CD513B-U6-*shEif2s3y* (*shEif2s3y*), and assistant plasmids PAX2 and VSVG were stored in Shaanxi Centre of Stem Cells Engineering & Technology, Northwest A&F University [27].

### 2.5. Lentivirus Preparation and Infection

Lentivirus production was described previously [28]. Assistant plasmids PAX2 and VSVG were cotransfected with pCDH-CMV-*Eif2s3y*-EF1-puro or CD513B-U6-*shEif2s3y* in HEK293T cells. The *oeEif2s3y* or *shEif2s3y* lentivirus was collected 48 hours later after substituted. The primary SSCs were infected with lentivirus *oeEif2s3y* or *shEif2s3y* when the density reached 80% complementing with polybrene (Sigma-Aldrich by Merck) to increase transfection efficiency. The infected SSCs were then cultured with a medium containing 500 ng/mL puromycin (Sigma-Aldrich by Merck) for 1 week in order to increase the proportion of positive cells.

### 2.6. Ethynyl-Deoxyuridine (EdU) Incorporation Assay

EdU incorporation assay was performed as per the manufacturer's instructions (C10310-1, RiboBio, Guangzhou, China). SSCs planted in a 48-well plate were incubated with the 50 *μ*M EdU medium for 2 h. Then, the EdU medium was discarded. Cells were fixed with 4% paraformaldehyde at room temperature for 15 min and decolorized in 2 mg/mL glycine for 10 min. After washing with PBS, cells were permeated by 0.5% Triton X-100 for 10 min. The staining buffer was added and incubated in the dark at room temperature for 30 min. After washing with PBS, the nuclei were visualized by Hoechst 33342 (Sigma-Aldrich by Merck). The cells were washed three times and observed under a fluorescence microscope.

Three culture wells were used in each group. At least three cell images and 300 cells per well were taken randomly. The ratio of the number of red fluorescent cells to the number of blue fluorescent cells is the ratio of positive cells [28]. The proportion of positive cells is positively correlated with the cell proliferation rate.

### 2.7. Quantitative Reverse Transcription-Polymerase Chain Reaction (qRT-PCR) Analysis

The qRT-PCR analysis was in accordance with a previous report [17]. Tissues and cells were harvested at the proper time, and total RNAs were extracted using the TRIzol reagent (RNAiso Plus, #9109, Takara Bio Inc., Japan). RNA integrity was analyzed by agarose gel electrophoresis, and the concentration was determined using a NanoDrop 2000 Spectrophotometer (Thermo Fisher Scientific, USA). Reverse transcription was performed using the RevertAid First Strand cDNA Synthesis Kit (Lot 00887496, Thermo Fisher Scientific, Waltham, Massachusetts, USA). Aliquots of undiluted cDNA were stored at -20°C and used for RT-PCR and real-time PCR. RT-qPCR was conducted on a CFX Connect Real-Time System (Bio-Rad, California, USA) using the SYBR Premix Real-Time PCR Kit (FP215-01, Tiangen Biotech, Beijing, China) in accordance with the manufacturer's instructions. The expression levels of mRNAs were normalized to GAPDH and *β*-actin. The qRT-PCR primers used in this article are listed in Supplemental Table 1. All primer sequences were determined through established GenBank sequences. The PCR efficiency was evaluated and analyzed by agarose gel electrophoresis.

### 2.8. Immunofluorescence (IF) Staining

Immunofluorescence staining of testes and SSCs was conducted as previously reported [27]. The primary antibodies used in this study are listed as follows: rabbit anti-eIF2*γ* (1 : 200; PA5-31177, Thermo Fisher Scientific, MA, USA), rabbit anti-DDX4 (1 : 200; ab13840, Abcam, Cambridge, UK), mouse anti-ZBTB16 (1 : 200; sc-28319, Santa Cruz Biotechnology, CA, USA), rabbit anti-STRA8 (1 : 200; ab49602, Abcam, Cambridge, UK), mouse anti-GFRa1 (1 : 200; sc-271546, Santa Cruz Biotechnology, CA, USA), rabbit anti-SOX9 (1 : 200; ab185230, Abcam, Cambridge, UK), and rabbit anti-StAR (1 : 100; bs-20388R, Bioss, Beijing, China). Secondary antibodies are as follows: Alexa Fluor 488-goat anti-rabbit IgG (1 : 400; ZF-0511, ZSGB-BIO, Beijing, China) and Alexa Fluor 568-goat anti-mouse (1 : 400; ZF-0513, ZSGB-BIO, Beijing, China).

### 2.9. Population Doubling Time (PDT) Determination

The PDT of dairy goat SSCs was estimated according to the protocol described previously [29]. Briefly, cells were serially subcultured; the initial seeding cell number and the total cell number cultured 24 h later were all counted, respectively. PDT was calculated according to the formula PDT = [log_2_/(log *N*_*t*_ − log *N*_0_)] × *t*, where *N*_0_ means the number of seeded cells, *N*_*t*_ indicates the number of cells after *t* (h) of culturing, and *t* means the duration of cell culturing hours.

### 2.10. Western Blotting

Western blotting (WB) was estimated according to a previous article [27]. The antibodies used in this study are listed as follows: anti-eIF2*γ* (1 : 500; PA5-31177, Thermo Fisher Scientific, MA, USA), anti-PCNA (1 : 500; BM0104, Boster, Wuhan, China), anti-Cyclin D (1 : 500; WL01435a, Wanlei, Shenyang, China), anti-GAPDH (1 : 2000; AC002, ABclonal, Wuhan, China), anti-ZBTB16 (1 : 500; No. D222893, BBI Life, Shanghai, China), anti-pERK (1 : 2000; #4370, CST, Boston, USA), and anti-ERK (1 : 2000; #9194, CST, Boston, USA). The results were detected using a Bio-Rad imaging system (Bio-Rad, Hercules, CA, USA) and quantified using ImageJ (V1.48d).

### 2.11. Bioinformatics Analysis

The dairy goat *Eif2s3y* CDS was sequenced by Sangon, China. Multiple sequence alignment among different species was performed by DNAMAN software, and the phylogenetic tree was depicted with MEGA 4.1. The amino acid sequences of *Eif2s3y* proteins in different species were also analyzed by DNAMAN software. The protein secondary structure was predicted by DNAStar software. The domains contained in *Eif2s3y* protein were predicted by the SWISS-MODEL Workspace website and RasMol software [30].

### 2.12. ERK Pathway Inhibitor and Activator

To confirm the function of ERK signaling in *Eif2s3y* regulation, SSCs were treated with 1 *μ*M ERK pathway inhibitor PD0325901 (APExBIO Technology LLC, A3013, Houston, USA) or 10 *μ*M ERK pathway activator 12-O-tetradecanoylphorbol-13-acetate (TPA, APExBIO Technology LLC, N2060, Houston, USA) for 24 h, respectively [31]. PD0325901 effectively inhibits the phosphorylation of ERK1/2 in multiple cell lines, while TPA activates the phosphorylation of ERK1/2 [32]. There were three replicates in each group of cells. The diluent of the reagent is DMSO.

### 2.13. Statistical Analysis

Relative gene expression was analyzed by the comparative Ct method (2^-*ΔΔ*Ct^ method). To compare significant differences, a two-tailed Student's *t*-test was used. The results were represented as mean ± SD. All results were replicated at least 3 times. Statistical analyses were analyzed by SPSS 20.0 software and GraphPad Prism software (La Jolla, CA). *P* values < 0.05 were considered statistically significant (^∗^*P* < 0.05, ^∗∗^*P* < 0.01, and ^∗∗∗^*P* < 0.001).

## 3. Results

### 3.1. The Expression Pattern of *Eif2s3y* in Dairy Goats

**Figure 1:**
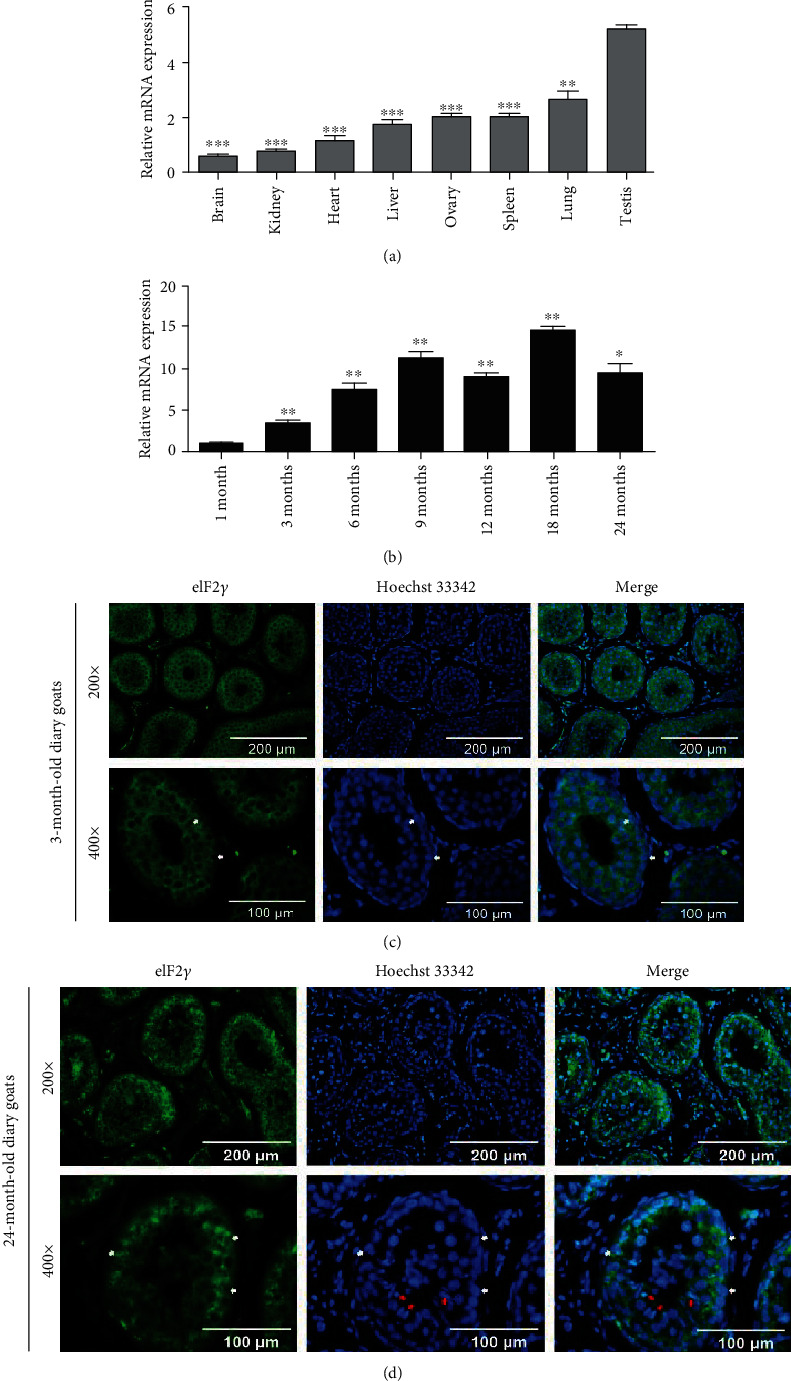
The expression pattern of *Eif2s3y* in dairy goats. (a) Real-time PCR analysis of *Eif2s3y* expression levels in different tissues of adult dairy goats. All results were compared with the testis. (b) Real-time PCR analysis of *Eif2s3y* expression levels in the testes of dairy goats of different ages. All results were compared with that of 1-month-old goats. (c) Immunofluorescence staining of eIF2*γ* in the testes of 3-month-old dairy goats counterstained with Hoechst 33342. The white arrows indicated typical SSCs. Scale bars, 200 *μ*m (up) and 100 *μ*m (down). (d) Immunofluorescence staining of eIF2*γ* in the testes of 24-month-old dairy goats counterstained with Hoechst 33342. The white arrows indicated typical SSCs, and the red arrows indicated typical sperms. Scale bars, 200 *μ*m (up) and 100 *μ*m (down). Data are presented as mean ± SD and are represented by three independent repetitions; ^∗^*P* < 0.05, ^∗∗^*P* < 0.01, and ^∗∗∗^*P* < 0.001.

First, we performed qRT-PCR to clarify the expression pattern of *Eif2s3y* in dairy goats. The results showed that *Eif2s3y* was widely expressed in different tissues including the brain, kidney, heart, liver, ovary, spleen, lung, and testis (Figure 1(a)). Of note, the testis showed the highest expression level of *Eif2s3y* (*P* < 0.01) and this level tended to increase gradually over time (*P* < 0.05), and it sustained high levels after sexual maturity than before (Figure 1(b)). Then, immunofluorescence staining was performed to analyze the expression pattern of eIF2*γ* in the testes of 3- and 24-month-old goats. The result showed that *Eif2s3y* could be expressed in SSCs, Sertoli cells, and Leydig cells (Figure 1(c); Supplemental Fig. 2A and B). Of note, lots of sperms could be observed in the testes of 24-month-old goats while not in those of 3-month-old goats (Figure 1(d); Supplemental Fig. 2C). We could see from these results that *Eif2s3y* was highly expressed in testes and eIF2*γ* protein mainly existed in the cytoplasm. The high expression of *Eif2s3y* in spermatogonia made us want to study their function in these cells.

### 3.2. Structure and Bioinformatics Analysis of *Eif2s3y* in Dairy Goats

**Figure 2:**
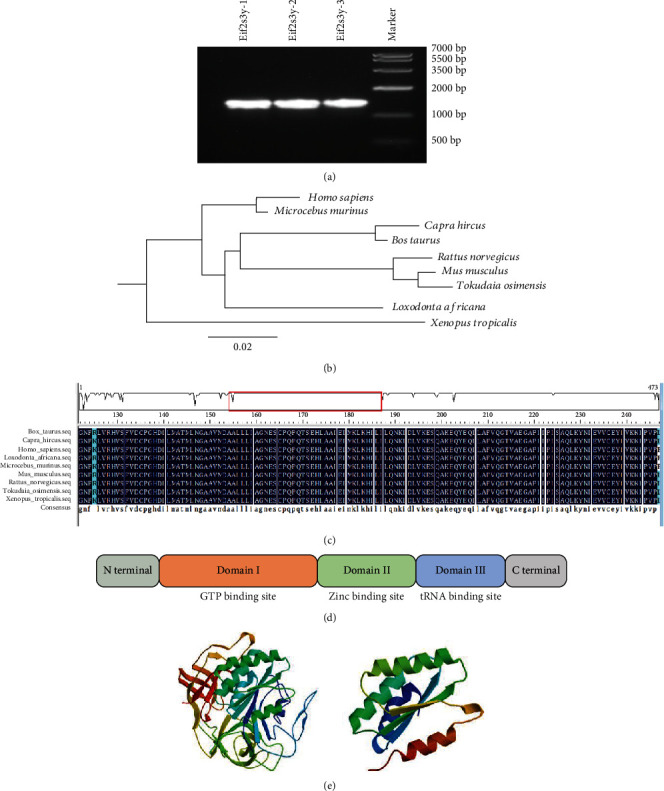
Structure and bioinformatics analysis of *Eif2s3y*. (a) PCR analysis of *Eif2s3y* in dairy goat testes. Three independent duplications all had the same size of 1413 bp. (b) *Eif2s3y* phylogenetic tree in different species constructed by MEGA 4.1 software. (c) *Eif2s3y* amino acid sequence alignment of different species analyzed by DNAMAN software. (d) Conserved domains of *Capra hircus Eif2s3y* CDS were predicted by NCBI CD-Search. Some important structures were found in conserved domains. (e) Eif2*γ* protein structural pattern (up) and four zinc finger domains (down) analyzed by the SWISS-MODEL Workspace website.

A pair of specific cloning primers for the CDS region of the dairy goat *Eif2s3y* gene was designed as described in Materials and Methods. We further cloned the *Eif2s3y* gene of dairy goats by PCR, and three repetitions were made (Figure 2(a)). The fragments whose sizes were between 1000 bp and 2000 bp were considered to be the goat *Eif2s3y* gene. Next, we inserted the gene into the pMD18-T vector for sequencing analysis, which showed that the size of the CDS region of the dairy goat *Eif2s3y* gene was 1413 bp. We uploaded the sequence information to the National Center for Biotechnology Information (NCBI) and obtained a formal gene serial number (GenBank: KP326346.1).

*Eif2s3y* gene sequences of *Homo sapiens*, *Microcebus murinus*, *Capra hircus*, *Bos taurus*, *Rattus norvegicus*, *Mus musculus*, *Tokudaia osimensis*, *Loxodonta africana*, and *Xenopus tropicalis* obtained from NCBI indicated that this gene was widely expressed in different species (Figure 2(b)). We analyzed the phylogenetic tree of *Eif2s3y* and compared their nucleotide and amino acid sequences (Figure 2(b)). The results showed a 97.98% similarity for amino acid among different species and suggested that *Eif2s3y* was highly conserved among different species (Figure 2(c)). Then, we predicted the protein structure of *Eif2s3y* through SWISS-MODEL Workspace and found an important binding region comprising four tandem zinc finger domains (Figure 2(e)). Another prediction gave a schematic map of protein domain analysis of Eif2*γ* by NCBI CD-Search (Figures 2(d) and 2(e)). These results indicated that *Eif2s3y* was conserved and might have similar functions in different species.

### 3.3. Overexpression of *Eif2s3y* Promotes the Proliferation of Dairy Goat SSCs

**Figure 3:**
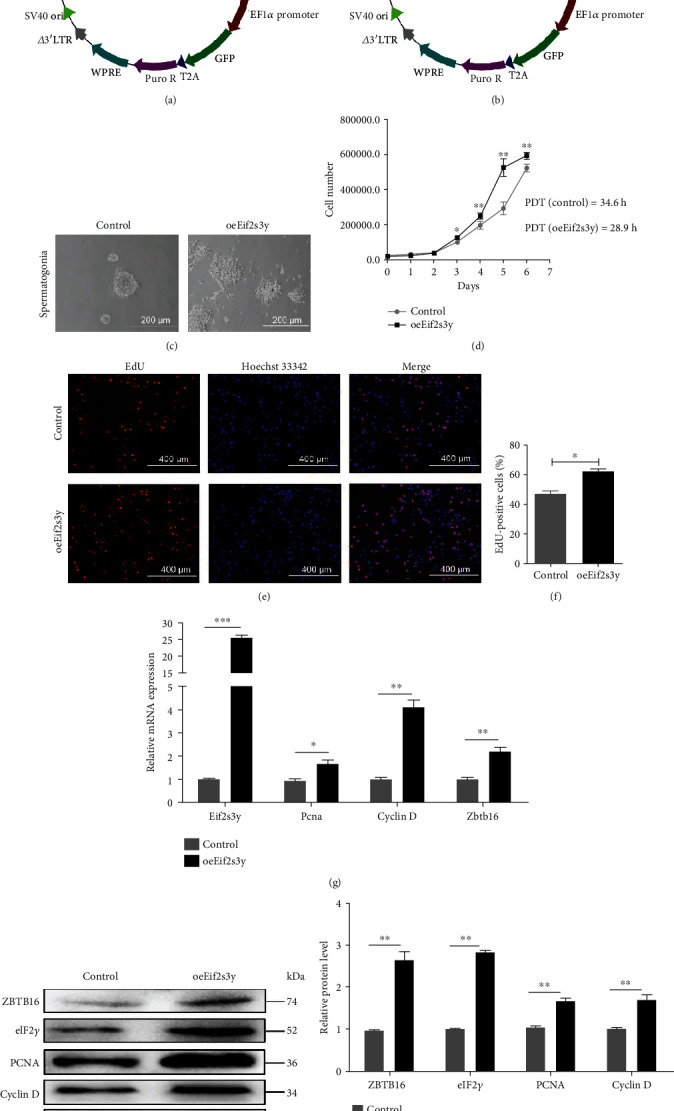
Overexpression of *Eif2s3y* promotes the proliferation of dairy goat SSCs. (a, b) The schematic of lentivirus plasmid pCDH-CMV-MCS-EF1-puro and pCDH-CMV-*Eif2s3y*-EF1-puro. (c) Typical images of SSCs transfected with Control-Vector (left) or *oeEif2s3y*-Vector (right). Scale bar, 200 *μ*m. (d) Proliferation curve of Control and *oeEif2s3y* SSCs and the results of population doubling time (PDT) determination. (e) EdU incorporation assay of Control (up) and *oeEif2s3y* (down) SSCs. Scale bar, 400 *μ*m. (f) The ratio of EdU-positive cells to total cells. The proportion of positive cells is positively correlated with the cell proliferation rate. (g) RT-PCR analysis of the expression levels of *Eif2s3y*, *Pcna*, Cyclin D, and *Zbtb16* in dairy goat SSCs transfected with Control-Vector or *oeEif2s3y*-Vector. (h) Western blotting detected the protein expression of ZBTB16, eIF2*γ*, PCNA, and Cyclin D in Control and *oeEif2s3y* SSCs. GAPDH was used as a loading control. (i) Gray intensity analysis of WB results normalized to GAPDH in (h). Data are presented as mean ± SD and are represented by three independent repetitions; ^∗^*P* < 0.05, ^∗∗^*P* < 0.01, and ^∗∗∗^*P* < 0.001.

The *Eif2s3y* fragment was inserted into the pCDH-CMV-MCS-EF1-puro vector, and a recombinant plasmid pCDH-CMV-*Eif2s3y*-EF1-puro was successfully constructed (Figures 3(a) and 3(b)). The pCDH-*Eif2s3y* and pCDH lentivirus were collected as described in Materials and Methods. The morphology and function of primary SSCs that we used to verify the direct effects of *Eif2s3y* have been verified in the past experimental studies [19, 21]. The primary cells and pure spermatogonia are shown in Supplemental Figure 1A. We examined the expression of several marker genes of SSCs by qRT-PCR and IF staining. The expression of SSC marker genes *Zbtb16*, *GFRa1*, and *Stra8* was significantly higher in the pure spermatogonia (Supplemental Figure 1B). The same conclusion was obtained by immunofluorescence staining (Supplemental Figure 1C). We successfully enriched SSCs.

The SSCs were infected with lentivirus pCDH-*Eif2s3y* or pCDH. After screening by 500 ng/mL puromycin (Sigma) for one week, *oeEif2s3y* cells and control cells were established (Figure 3(c)). Interestingly, the morphology of *oeEif2s3y* cells changed and the edge of colonies became unsmooth, showing a certain extent of differentiation. According to a previous report, the *Eif2s3y* defect would block the production of spermatogonia and result in infertility in mice [13]. Our results showed that *Eif2s3y* promoted the proliferation of goat SSCs, as reflected by the higher proliferation rate of *oeEif2s3y* cells in cell number counting (Figure 3(d)). The population doubling time (PDT) of *oeEif2s3y* cells was significantly reduced from 34.6 hours to 28.9 hours. The results were further strengthened by EdU incorporation assay (Figures 3(e) and 3(f)). In accordance with these findings, we found that the expression levels of proliferation-associated genes (*Pcna*, Cyclin D) and self-renewal-associated gene (*Zbtb16*) were increased in *oeEif2s3y* cells (Figure 3(g)). The proportion of red positive cells was consistent with the cell proliferation rate. Western blotting was applied to exam the effect of transgenic *Eif2s3y* (Figure 3(h)). In *oeEif2s3y* SSCs, statistical analysis showed that the expression of ZBTB16, eIF2*γ*, Pcna, Cyclin D was higher than in Control SSCs (Figure 3(i)). Collectively, these data demonstrated that overexpression of *Eif2s3y* promoted the proliferation of goat SSCs.

### 3.4. *Eif2s3y* Deficiency Reversed the Goat SSC Growth Rate

**Figure 4:**
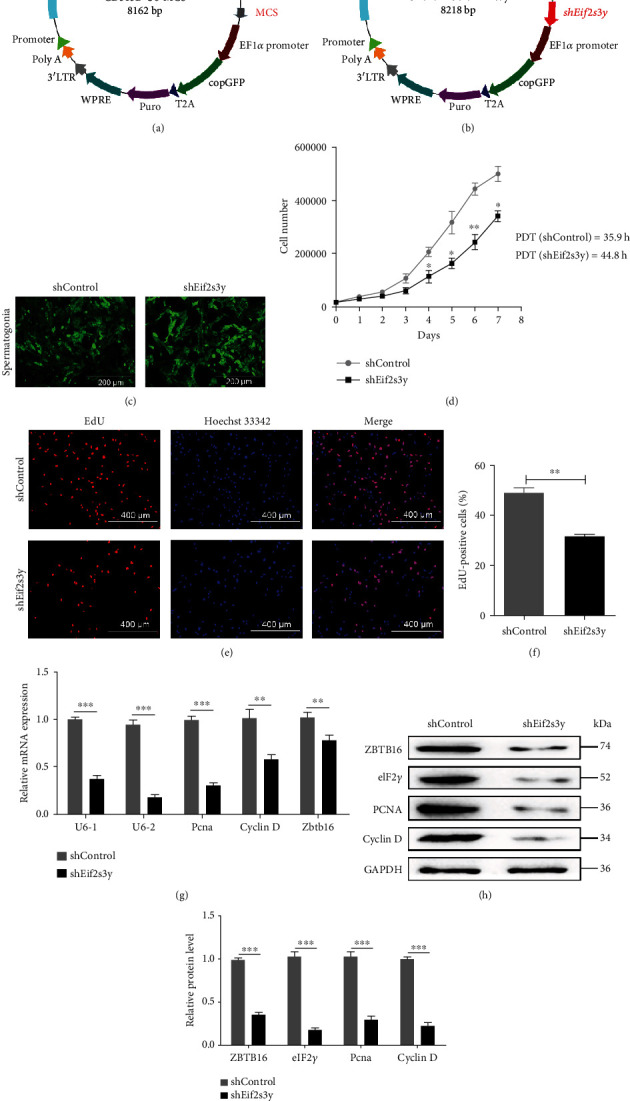
Depletion of *Eif2s3y* resulted in proliferation abnormality in goat SSCs. (a, b) The schematic of lentivirus plasmid CD513B-U6-MCS and CD513B-U6-*shEif2s3y*. (c) Typical images of *shControl* (left) and *shEif2s3y* (right) SSCs. Scale bar, 200 *μ*m. (d) Proliferation curve of *shControl* and *shEif2s3y* SSCs and the results of population doubling time (PDT) determination. (e) EdU incorporation assay of *shControl* (up) and *shEif2s3y* (down) SSCs. Scale bar, 400 *μ*m. (f) The ratio of EdU-positive cells to total cells. Data are presented as mean ± SD and are represented by three independent repetitions. (g) RT-PCR analysis of the expression levels of *Eif2s3y*, *Pcna*, Cyclin D, and *Zbtb16* in *shControl* and *shEif2s3y* SSCs *in vitro*. (h) Western blotting detected the protein expression of ZBTB16, eIF2*γ*, PCNA, and Cyclin D in *shControl* and *shEif2s3y* SSCs. GAPDH was used as a loading control. (i) Gray intensity analysis of WB results normalized to GAPDH in (h). Data are presented as mean ± SD and are represented by three independent repetitions; ^∗^*P* < 0.05, ^∗∗^*P* < 0.01, and ^∗∗∗^*P* < 0.001.

Since overexpression of *Eif2s3y* could promote SSC proliferation, we wondered whether knockdown of *Eif2s3y* expression would inhibit this proliferation. Recombinant plasmid CD513B-U6-*shEif2s3y* was successfully constructed (Figures 4(a) and 4(b)). Seven days after infection with lentivirus, RT-PCR analysis confirmed the successful knocking down of *Eif2s3y* expression (*shEif2s3y*) in goat SSCs. The efficiency of the two interfering fragments was 60% or 90%, respectively (Figure 4(g)). We chose the more efficient U6-Vector2 for future experiments (Figures 4(c) and 4(e)).

The population doubling time of *shControl* and *shEif2s3y* SSCs was 35.9 or 44.8 hours, respectively (Figure 4(d)). Then, we evaluated the proliferation rate by EdU staining; the percentage of EdU-positive *shEif2s3y* cells was lower than that of *shControl* (Figures 4(e) and 4(f)). Compared with the *shControl* group, the expression levels of proliferation-associated genes *Pcna* and Cyclin D and self-renewal-associated gene *Zbtb16* in the *shEif2s3y* group were significantly decreased (Figures 4(g) and 4(h)). Western blotting analysis got the same results (Figure 4(i)). These experiments showed that *Eif2s3y* deficiency reversed goat SSC proliferation.

### 3.5. *Eif2s3y* Could Increase the Colonization Rate of Goat SSCs in SSCT

**Figure 5:**
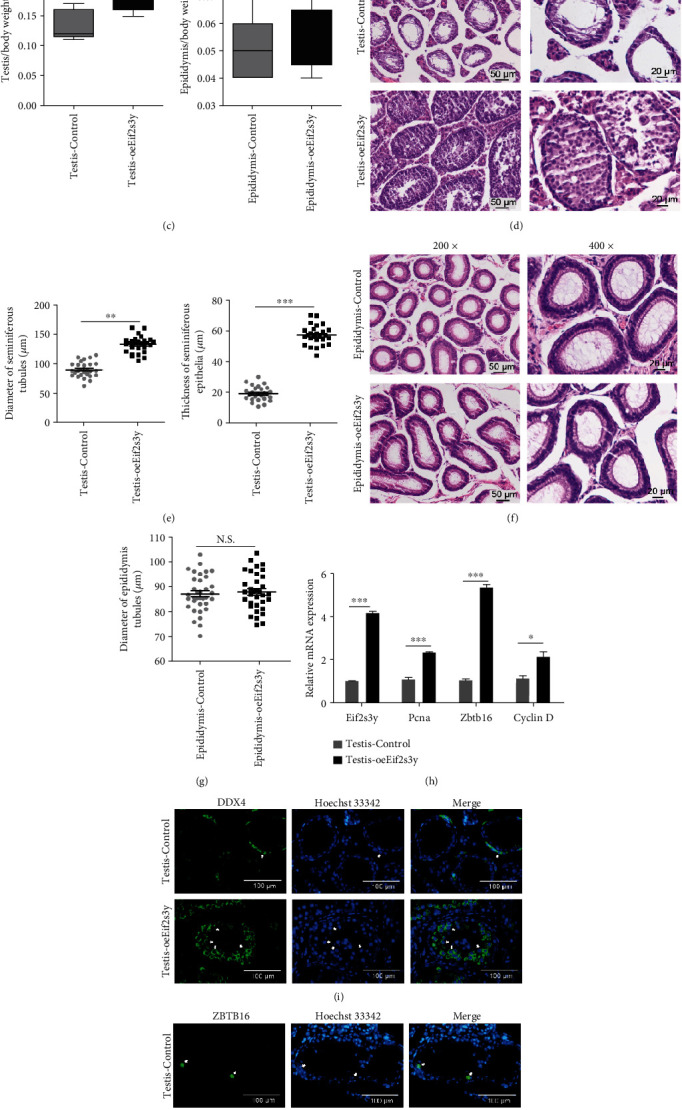
*Eif2s3y* promotes the colonization of goat SSCs in SSCT. (a) *OeEif2s3y* or *Control* SSCs were injected into the seminiferous tubules of busulfan-treated mice. The figure here shows two typical pictures of the testis during injection. Scale bars, 1 mm and 5 mm as indicated. (b) Morphology of testes and epididymides transfected with *oeEif2s3y* or *Control* SSCs. (c) The testicular (left) and epididymis (right) weight/body mass ratio in two groups. (d) H&E staining of the mouse testis 30 days after injection. Scale bars, 50 *μ*m (left) and 20 *μ*m (right). (e) Statistical plots of the diameter of seminiferous tubules (left) and the thickness of seminiferous epithelia (right) from *oeEif2s3y* and *Control* SSC-transplanted mice. Each group counted at least 30 round seminiferous tubules from 10 mice. (f) H&E staining of the mouse epididymis 30 days after injection. Scale bars, 50 *μ*m (left) and 20 *μ*m (right). (g) Statistical plots of the diameter of epididymis tubules from *oeEif2s3y* and *Control* SSC-transplanted mice. Each group counted at least 30 epididymis tubules from 10 mice. (h) RT-PCR analysis of the expression of *Eif2s3y*, *Pcna*, *Zbtb16*, and Cyclin D in Testis-Control and Testis-*oeEif2s3y*. (i) Immunofluorescence staining of DDX4 (green) in SSC-transplanted testes. The nuclei were stained with Hoechst 33342 (blue). Scale bar, 100 *μ*m. These white arrows represented typical DDX4-positive germ cells. DDX4 is a representative marker for pan-germ cells. (j) Immunofluorescence staining of ZBTB16 (green) in SSC-transplanted testes. The nuclei were stained with Hoechst 33342 (blue). Scale bar, 100 *μ*m. These white arrows represented typical ZBTB16-positive spermatogonial stem cells. ZBTB16 is a representative marker for undifferentiated spermatogonia. Data are presented as mean ± SD and are represented by three independent repetitions; ^∗^*P* < 0.05, ^∗∗^*P* < 0.01, and ^∗∗∗^*P* < 0.001; N.S. means *P* ≥ 0.05.

**Figure 6:**
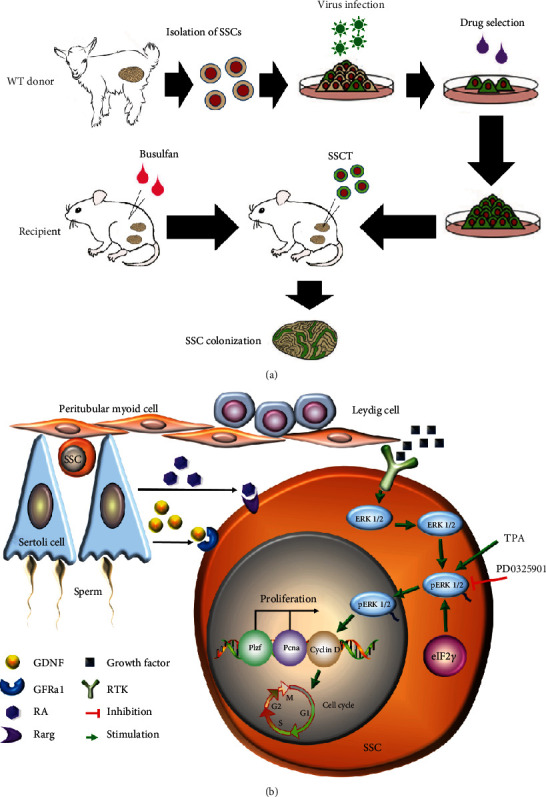
A schematic diagram illustrating *Eif2s3y* functions in goat SSCs. (a) Schematic diagram of seminiferous tubule transplantation. (b) A proposed model for *Eif2s3y* activating the downstream ERK signaling pathway to regulate proliferation genes in goat SSCs. This finding may provide an important basis for future works regarding male spermatogenic cell restoration in large animals.

Spermatogonial stem cell transplantation technology has been an effective method to study SSCs since 1994 [1, 26, 33]. Some previous research had proved the reliability of our transplantation technique [17]. To investigate the contribution of *Eif2s3y* in SSCs, *oeEif2s3y* SSCs and *Control* SSCs were transferred into the seminiferous tubules of twenty infertile mice treated with busulfan (Figures 5(a) and 6(a)). Testes transplanted with *oeEif2s3y* SSCs were heavier than those in the control group (*P* = 0.014), while the weight of the epididymis did not change significantly (*P* = 0.43) (Figures 5(b) and 5(c)).

H&E staining of the transplanted testes showed that more germ cells were observed in the *Eif2s3y* group (Figure 5(d)). Additionally, the diameter of seminiferous tubules (*P* < 0.01) and the thickness (*P* < 0.001) of seminiferous epithelia were significantly increased in the *Eif2s3y* group (Figure 5(e)). However, the diameter of epididymis tubules was not significant between these two groups (Figures 5(f) and 5(g)). In addition, RT-PCR analysis showed that *Eif2s3y*, *Pcna*, *Zbtb16*, and Cyclin D were overexpressed in the *oeEif2s3y* group (Figure 5(h)). Importantly, immunofluorescence staining showed that the *Eif2s3y* group had more DDX4-positive germ cells and more ZBTB16-positive SSCs in the testes (Figures 5(i) and 5(j)). Thus, overexpression of *Eif2s3y* might contribute to improving the survival rate and proliferation of goat SSCs in SSCT. However, no sperm was found in either group, which might be caused by different species (Figure 5(d)).

### 3.6. *Eif2s3y* Promotes the Proliferation of SSCs by Activating the ERK Signaling Pathway

**Figure 7:**
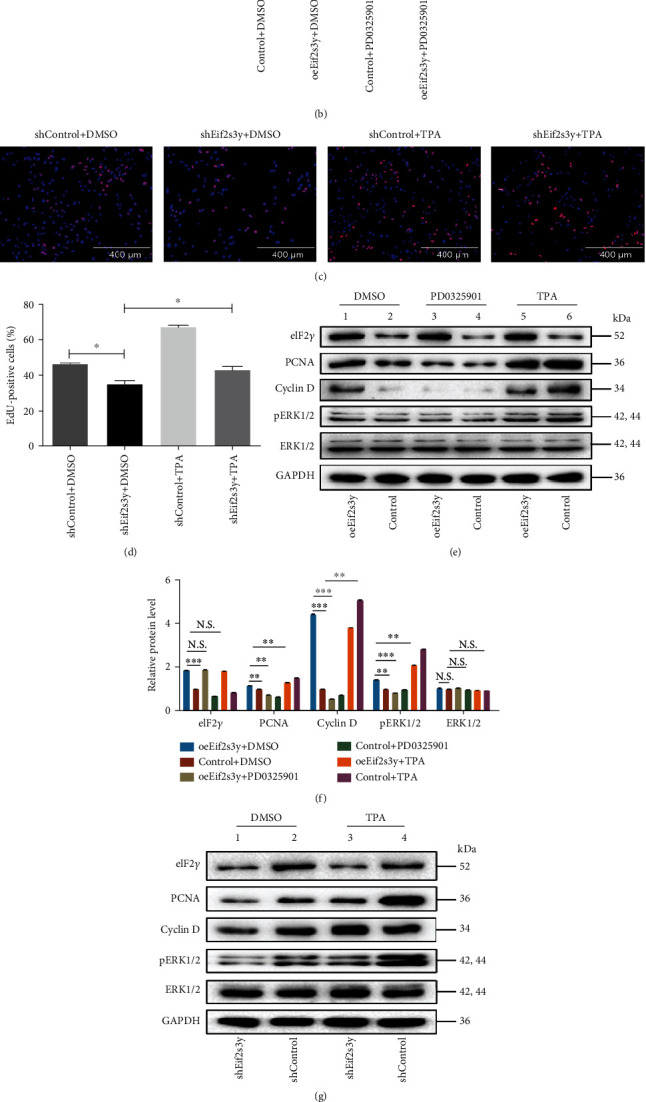
*Eif2s3y* promotes SSC proliferation by activating the ERK signaling pathway. *Control* and *oeEif2s3y* SSCs were treated with 1 *μ*M ERK pathway inhibitor PD0325901 or 10 *μ*M ERK pathway activator TPA for 24 h, respectively. *shControl* and *shEif2s3y* SSCs were treated with 10 *μ*M ERK pathway activator TPA for 24 h. DMSO was used for the control group. (a) EdU incorporation assay of *Control* and *oeEif2s3y* SSCs added with ERK pathway inhibitor PD0325901 or DMSO. Scale bar, 400 *μ*m. (b) The percentage of EdU-positive cells to total cells. The proportion of positive cells is positively correlated with the cell proliferation rate. (c) EdU incorporation assay of *shControl* and *shEif2s3y* SSCs added with ERK pathway activator TPA or DMSO. Scale bar, 400 *μ*m. (d) The percentage of EdU-positive cells to total cells. (e) Western blotting detected the protein expression of eIF2*γ*, PCNA, Cyclin D, pERK1/2, and ERK1/2 in SSCs treated as indicated. GAPDH was used as a loading control. (f) Gray intensity analysis of WB results normalized to GAPDH in (e). (g) Western blotting detected the protein expression of eIF2*γ*, PCNA, Cyclin D, pERK1/2, and ERK1/2 in SSCs treated as indicated. (h) Gray intensity analysis of WB results normalized to GAPDH in (g). Data are presented as mean ± SD and are represented by three independent repetitions; ^∗^*P* < 0.05, ^∗∗^*P* < 0.01, and ^∗∗∗^*P* < 0.001; N.S. means *P* ≥ 0.05.

To confirm the proliferation mechanism of *Eif2s3y* in goat SSCs, we investigated the proliferation-related signaling pathways through western blotting in *oeEif2s3y* and *shEif2s3y* SSCs. Our previous study found that the expression of *Eif2s3y* increased the phosphorylation level of ERK. Then, SSCs were treated by 1 *μ*M ERK pathway inhibitor PD0325901 [31] or 10 *μ*M ERK pathway activator TPA [32] for 24 hours, respectively. DMSO was used for the control group. As expected, PD0325901 treatment significantly inhibited the proliferation of *oeEif2s3y* SSCs, while TPA treatment efficiently restored the proliferation of SSCs transplanted with *shEif2s3y*, as analyzed by EdU incorporation assay (Figures 7(a)–7(d)).

Western blotting analysis was further conducted to explore the underlying mechanisms, and we found that the MEK-ERK signal could directly and indirectly participate in the proliferation of SSCs. When ERK signaling was activated by *Eif2s3y* overexpression or by TPA treatment, the expression levels of PCNA and Cyclin D were both increased. The results showed that the ERK signaling blockade, either by *Eif2s3y* interference or by PD0325901 treatment, significantly inhibited the expression levels of PCNA and Cyclin D (Figures 7(e) and 7(f)). In comparison, the results showed that the ERK signaling blockaded, either by *Eif2s3y* interference or by PD0325901 treatment, significantly inhibited the expression levels of PCNA and Cyclin D, both of which were essentially needed during cell proliferation (Figures 7(g) and 7(h)). According to the above experimental results, we hypothesized that *Eif2s3y* activated the downstream ERK signaling pathway to regulate proliferation genes.

According to the above experimental results, we proposed a mechanism diagram of how *Eif2s3y* activated the downstream ERK signaling pathway to promote SSC proliferation and restoration of male spermatogenic cells in goat testes (Figure 6(b)).

## 4. Discussion

*Eif2s3y* is widely expressed in different male animals and recognized as a translation initiation factor [11, 34]. In recent studies, we found that *Eif2s3y* could regulate the proliferation of goat SSCs. The expression levels of proliferation- and self-renewal-related genes Cyclin D, *Cyclin A*, *Pcna*, and *Plzf* were upregulated in *oeEif2s3y* SSCs and downregulated in *shEif2s3y* SSCs (Figures 3(g) and 4(g)). Our results showed that *Eif2s3y* played an important role in male reproduction of dairy goats, and these results were consistent with previous studies in mice that *Eif2s3y* played a critical role in male spermatogenesis [14, 15, 35]. Importantly, we found a regulatory pathway of *Eif2s3y* in male reproduction with ERK signaling involved. This finding was in line with previous studies reported by us and other groups that the MEK/ERK signaling pathway played an important role in cell proliferation, differentiation, and cell cycle progression [36, 37]. However, how *Eif2s3y* regulates ERK signaling remains to be further studied.

SSCT technology is an effective method to identify the characteristics of SSCs cultured *in vitro* [1]. We transferred *oeEif2s3y* SSCs into the seminiferous tubules of infertile mice and found that *Eif2s3y* could enhance the colonization of germ cells (Figure 5(d)). Moreover, no mature sperm was observed in the epididymis (Figure 5(e)). The blood-testis barrier (BTB) made goat SSCs transplanted into mouse testes survive [5]. However, the species relationship between goats and mice was so far that mice could not produce goat sperms. As a contrast, we injected CD513B-U6-*shEif2s3y* lentivirus into the seminiferous tubules of wild-type mice and found that spermatogenesis was blocked and the germ cells in seminiferous tubules were very loose. Another research group directly knocked out the mouse *Eif2s3y* gene with TALEN technology, which led to testicular hypoplasia and male infertility [13]. More generally, all these results of animal experiments *in vivo* indicated that *Eif2s3y* played an important role in spermatogenesis.

Our bioinformatics analysis showed that *Eif2s3y* was highly conserved among different species (Figure 2(c)). Therefore, the interference experiment of mice might also be applicable to goats. Dairy goat *Eif2s3y* was located on the Y chromosome and encoded a 471 amino acid protein which contained a compact zinc finger domain and an N-terminal GTP binding domain (Figures 2(d) and 2(e)). In mice, *Eif2s3x* was a homologous gene of *Eif2s3y* and shared 98% of amino acid sequence identity and almost all of the RNA binding domains with *Eif2s3y* [35]. However, recent studies have found that these two genes might not completely replace each other. *Eif2s3x* has been found to play an irreplaceable role in the early development of organs such as the brain and pancreas [38–40]. Meanwhile, a recent study showed that *Eif2s3y* was more effective in masculinizing mice during sex growth at 12.5 days of mouse embryonic development [41]. However, in dairy goats, it was still unclear whether a homologous gene of *Eif2s3y* exists and how they worked together. Thus, future work is essentially needed to answer these questions.

In conclusion, our study found a novel role of *Eif2s3y* in the male reproduction of dairy goats. This finding might provide an important basis for the repair of male infertility and spermatogonial stem cell transplantation toward realizing the regeneration of germplasm in large animals.

## Data Availability

The data that support the findings of this study are available from the corresponding author upon reasonable request.

## Supplementary Materials

Supplementary 1 Supplemental Figure 1: isolation and enrichment of dairy goat spermatogonia. (A) The primary cells and pure spermatogonia isolated from healthy dairy goat testes; scale bar = 200 *μ*m. (B) RT-PCR analysis of the expression of *Zbtb16*, *GFRa1*, and *Stra8* in primary cells and pure spermatogonia. Data are presented as mean ± SD and are represented by three independent repetitions; ^∗^*P* < 0.05, ^∗∗^*P* < 0.01. (C) Immunofluorescence staining of ZBTB16 (up), STRA8 (middle), and GFRa1 (down) in pure spermatogonia. The nuclei were stained with Hoechst 33342 (blue). Scale bar, 200 *μ*m. ZBTB16, STRA8, and GFRa1 are representative markers for SSCs.

Supplementary 2 Supplemental Figure 2: some additional pictures. (A) Immunofluorescence staining of mouse (up) and goat (down) testes. Only IgG (1 : 200) was used for the first antibody, and the fluorescent secondary antibody was used normally. The nuclei were stained with Hoechst 33342 (blue). Scale bar, 200 *μ*m. Species-specific IgG antibody for immunofluorescence experiments as the parallel negative antigen control to prove that there was no false positive in our immunofluorescence staining. (B) Immunofluorescence staining of SOX9 (up) and StAR (down) in goat testes. The nuclei were stained with Hoechst 33342 (blue). Scale bar, 100 *μ*m. SOX9 is a representative marker for Sertoli cells, and StAR is a representative marker for Leydig cells. (C) H&E staining of 3-month-old and 24-month-old goat testes. Scale bars, 50 *μ*m (left) and 20 *μ*m (right).

Supplementary 3 Supplemental Figure 3: full unedited images. (A) Full unedited PCR images of Figure 2(a) data. (B) Full unedited western blotting images of Figure 7(e) data. (C) Full unedited western blotting images of Figure 7(g) data.

Supplementary 4 Supplemental Table 1: the sequence and length of primers used in qRT-PCR amplification.

Supplementary 5 Supplemental File 1: normal distribution test for experimental data.

Supplementary 6 Supplemental File 2: experimental information for qRT-PCR.
