# Supplementary material for: Eif2s3y Promotes the Proliferation of Spermatogonial Stem Cells by Activating ERK Signaling
Source: Stem Cells Int. 2021 Jan 29;2021:6668658. doi: 10.1155/2021/6668658 (PMC7869416; doi:10.1155/2021/6668658)
Supplement: Supplementary 1 — Supplemental Figure 1: isolation and enrichment of dairy goat spermatogonia. (A) The primary cells and pure spermatogonia isolated from healthy dairy goat testes; scale bar = 200 μm. (B) RT-PCR analysis of the expression of Zbtb16, GFRa1, and Stra8 in primary cells and pure spermatogonia. Data are presented as mean ± SD and are represented by three independent repetitions; ∗P < 0.05, ∗∗P < 0.01. (C) Immunofluorescence staining of ZBTB16 (up), STRA8 (middle), and GFRa1 (down) in pure spermatogonia. The nuclei were stained with Hoechst 33342 (blue). Scale bar, 200 μm. ZBTB16, STRA8, and GFRa1 are representative markers for SSCs. [file 6668658.f1.docx]

**
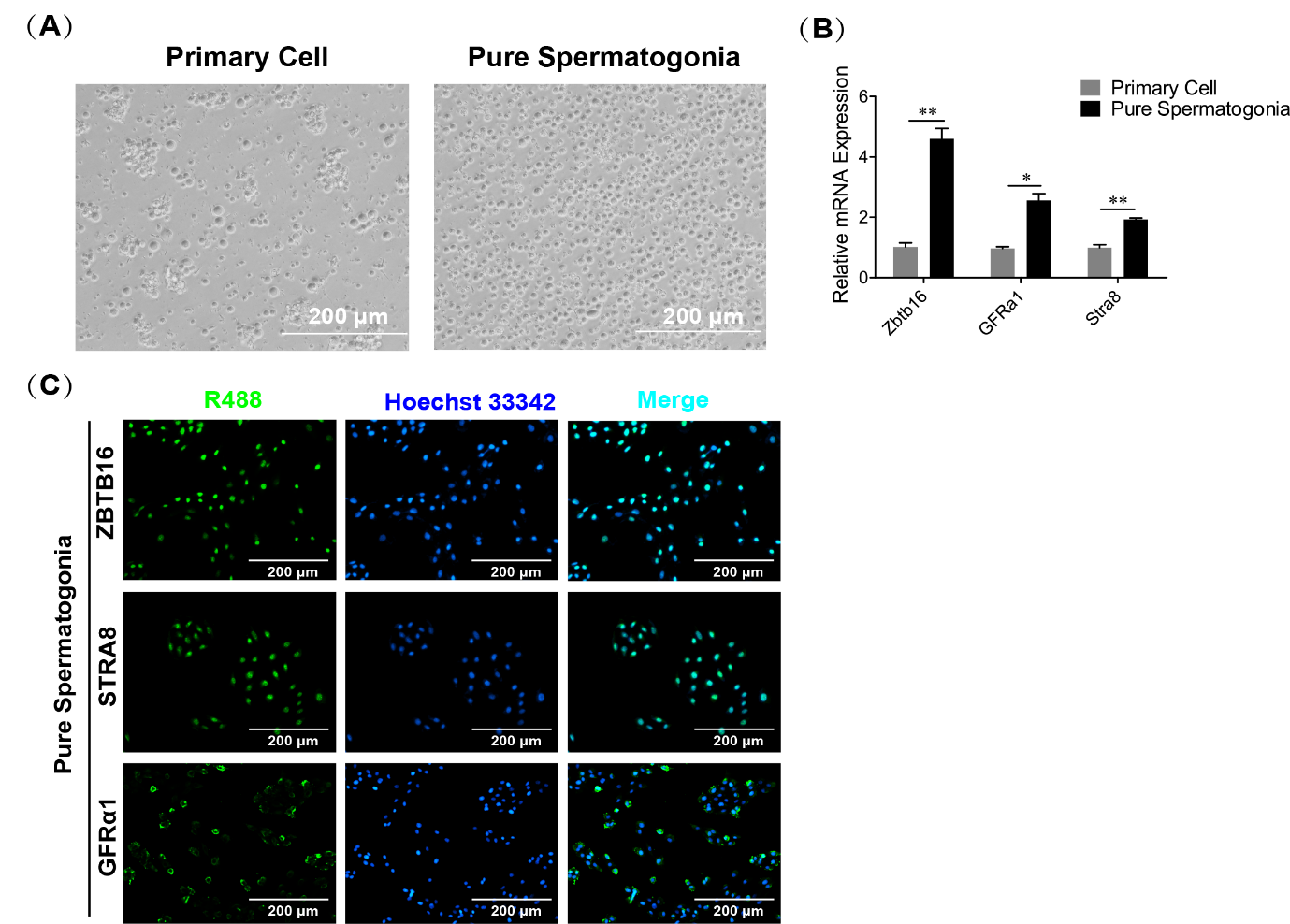
Supplemental Figure 1 Isolation and enrichment of dairy goat spermatogonia.**

(A) The primary cell and pure spermatogonia isolated from healthy dairy goat testis, scale bar = 200 μm. (B) RT-PCR analysis of the expression of *Zbtb16*, *GFRa1*, *Stra8* in primary cell and pure spermatogonia. Data are presented as means ± SD and are represented of three independent repetitions (*) *P* < 0.05, (**) *P* < 0.01. (C) Immunofluorescence staining of ZBTB16 (up), STRA8 (middle), GFRa1 (down) in pure spermatogonia. The nuclei were stained with Hoechst 33342 (blue). Scale bars, 200 μm. ZBTB16, STRA8 and GFRa1 are representative markers for SSCs.
