# Supplementary material for: Eif2s3y Promotes the Proliferation of Spermatogonial Stem Cells by Activating ERK Signaling
Source: Stem Cells Int. 2021 Jan 29;2021:6668658. doi: 10.1155/2021/6668658 (PMC7869416; doi:10.1155/2021/6668658)
Supplement: Supplementary 2 — Supplemental Figure 2: some additional pictures. (A) Immunofluorescence staining of mouse (up) and goat (down) testes. Only IgG (1 : 200) was used for the first antibody, and the fluorescent secondary antibody was used normally. The nuclei were stained with Hoechst 33342 (blue). Scale bar, 200 μm. Species-specific IgG antibody for immunofluorescence experiments as the parallel negative antigen control to prove that there was no false positive in our immunofluorescence staining. (B) Immunofluorescence staining of SOX9 (up) and StAR (down) in goat testes. The nuclei were stained with Hoechst 33342 (blue). Scale bar, 100 μm. SOX9 is a representative marker for Sertoli cells, and StAR is a representative marker for Leydig cells. (C) H&E staining of 3-month-old and 24-month-old goat testes. Scale bars, 50 μm (left) and 20 μm (right). [file 6668658.f2.docx]

**
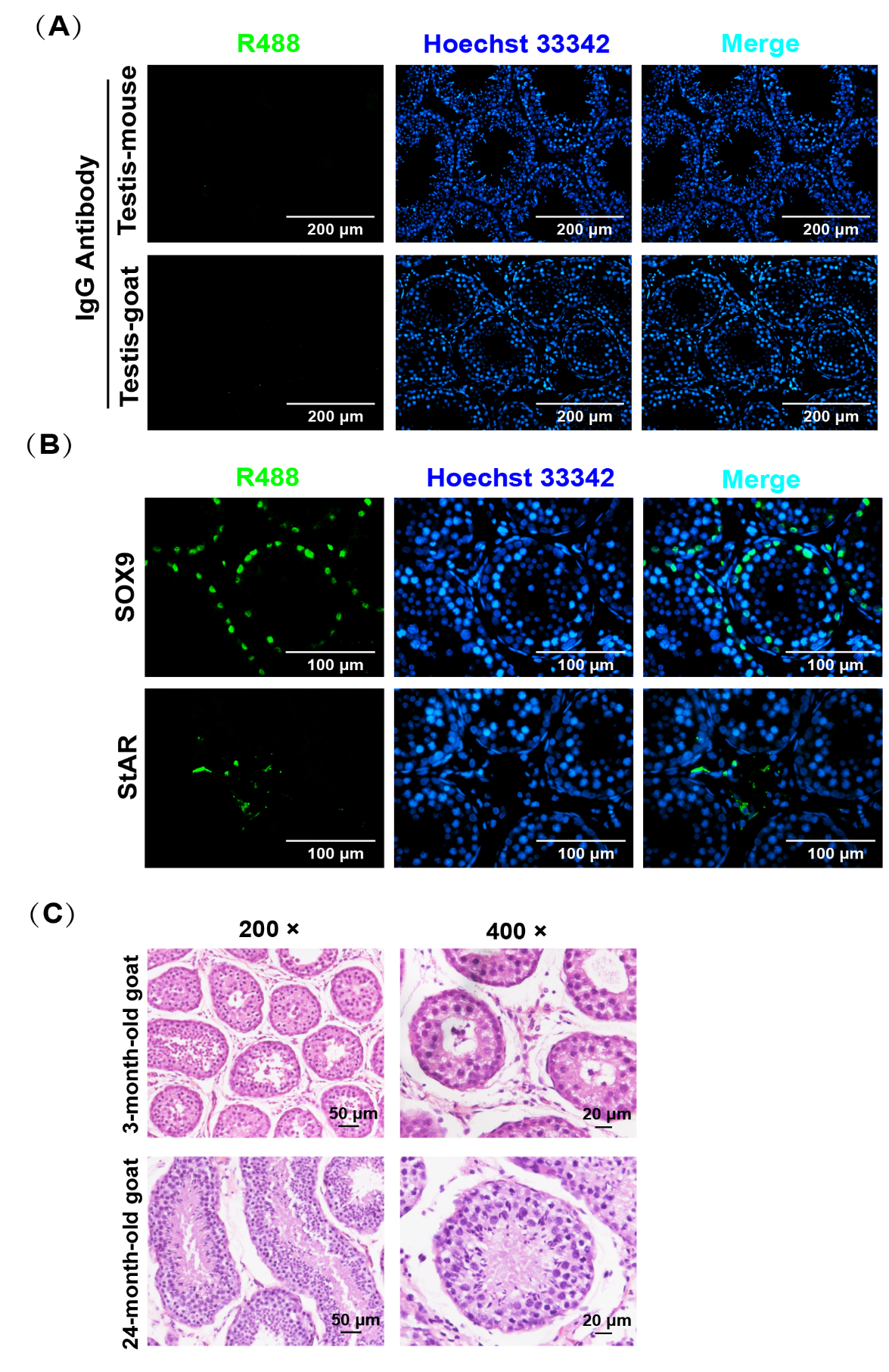
**

**Supplemental Figure 2 Some additional pictures.**

(A) Immunofluorescence staining of mouse (up) and goat (down) testis. Only IgG (1:200) was used for the first antibody and the fluorescent secondary antibody was used normally. The nuclei were stained with Hoechst 33342 (blue). Scale bars, 200 μm. Species-specific IgG antibody for Immunofluorescence experiments as the parallel negative antigen control to prove that there was no false positive in our immunofluorescence staining. (B) Immunofluorescence staining of SOX9 (up) and StAR (down) in goat testes. The nuclei were stained with Hoechst 33342 (blue). Scale bars, 100 μm. SOX9 is a representative marker for Sertoli cells and StAR is a representative marker for Leydig cells. (C) H&E staining of 3-month-old and 24-month-old goat testes. Scale bars, 50 μm (left) and 20 μm (right).
