# Supplementary material for: Eif2s3y Promotes the Proliferation of Spermatogonial Stem Cells by Activating ERK Signaling
Source: Stem Cells Int. 2021 Jan 29;2021:6668658. doi: 10.1155/2021/6668658 (PMC7869416; doi:10.1155/2021/6668658)
Supplement: Supplementary 3 — Supplemental Figure 3: full unedited images. (A) Full unedited PCR images of Figure 2(a) data. (B) Full unedited western blotting images of Figure 7(e) data. (C) Full unedited western blotting images of Figure 7(g) data. [file 6668658.f3.docx]

**
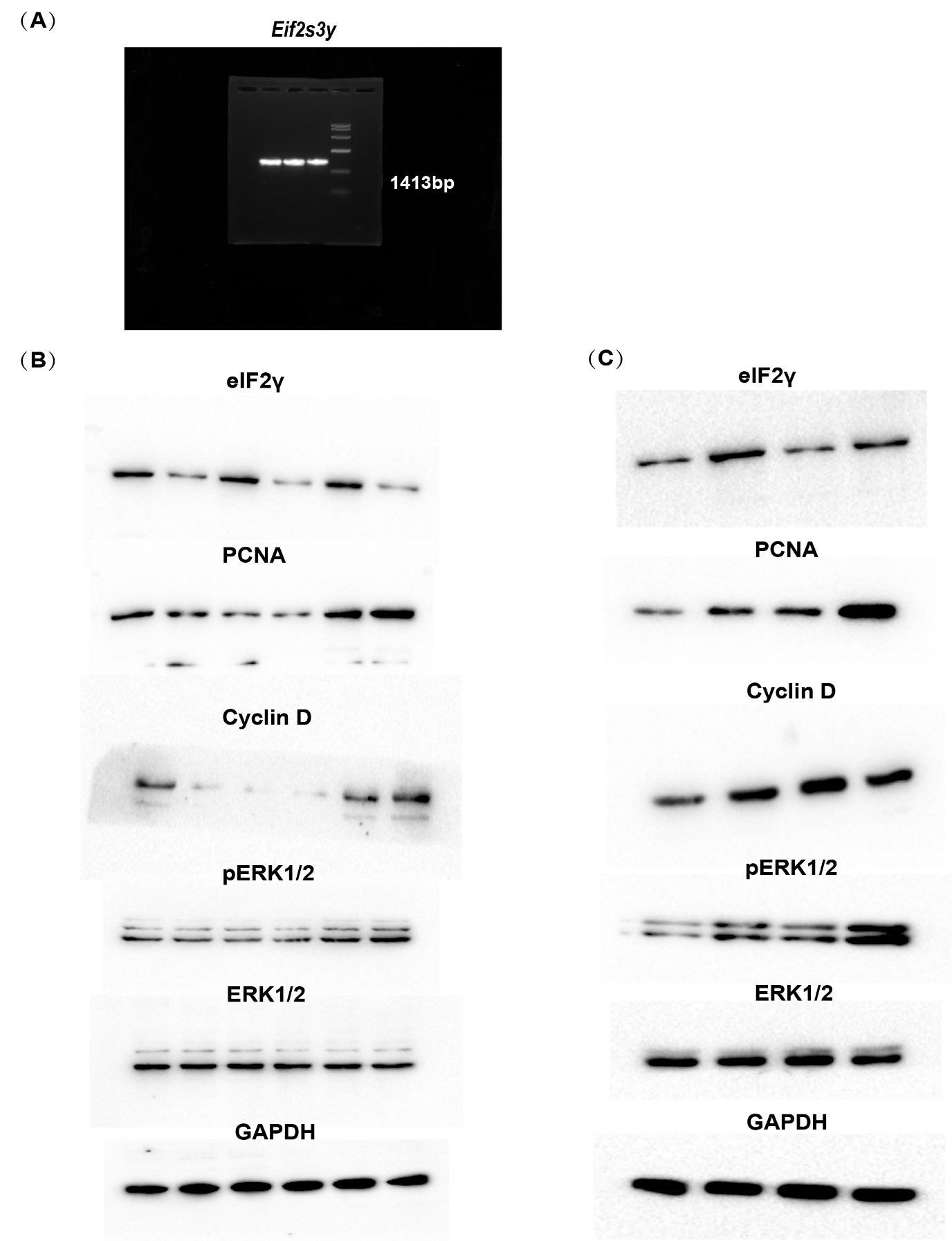
**

**Supplemental Figure 3 Full unedited images.**

(A) Full unedited PCR images of Figure 2A data. (B) Full unedited western blot images of Figure 6E data. (C) Full unedited western blot images of Figure 6G data.
