# Supplementary material for: Eif2s3y Promotes the Proliferation of Spermatogonial Stem Cells by Activating ERK Signaling
Source: Stem Cells Int. 2021 Jan 29;2021:6668658. doi: 10.1155/2021/6668658 (PMC7869416; doi:10.1155/2021/6668658)
Supplement: Supplementary 4 — Supplemental Table 1: the sequence and length of primers used in qRT-PCR amplification. [file 6668658.f4.docx]

**Supplemental Table 1 The sequence and length of primers used in qRT-PCR amplification.**

| Gene name | Sense primer sequence (5ʹ−3ʹ) | Antisense primer sequence (5ʹ−3ʹ) | Product size /bp | Tm/℃ | GenBank |
| --- | --- | --- | --- | --- | --- |
| *Ei2s3y* | CCTTTGCTGCTTTCTTGTCTCC | TGCTGCTCCAGGTGGTCTTATT | 194 | 60 | >XM_018044897.1 |
| *Cyclin D* | GCGTACCCTGACACCAATCTC | CTCCTCTTCGCACTTCTGCTC | 183 | 60 | >XM_018043271.1 |
| *Pcna* | AGTGGAGAACTTGGAAATGGAA | GAGACAGTGGAGTGGCTTTTGT | 154 | 60 | >XM_005688167.3 |
| *Zbtb16* | CACCGCAACAGCCAGCACTAT | CAGCGTACAGCAGGTCATCCAG | 127 | 60 | >XM_018058857.1 |
| *GFRα1* | GGACAGGCAGCAGGAAATA | GTCTCCTGTCCCAGTCAAA | 201 | 60 | >XM_018041626.1 |
| *Stra8* | AAGGACAGCGGGGTTGAC | TCGGGAGTTTTTGAGTTGC | 170 | 60 | >XM_018047489.1 |
| *β-actin* | TGATATTGCTGCGCTCGT | CTTGAGGGTCAGGATGCC | 196 | 60 | >XM_018039831.1 |
| *GAPDH* | CGTGTCCGTTGTGGATCTGA | TGAAGTCGCAGGAGACAACC | 143 | 60 | >XM_005680968.3 |
