# Supplementary material for: Eif2s3y Promotes the Proliferation of Spermatogonial Stem Cells by Activating ERK Signaling
Source: Stem Cells Int. 2021 Jan 29;2021:6668658. doi: 10.1155/2021/6668658 (PMC7869416; doi:10.1155/2021/6668658)
Supplement: Supplementary 5 — Supplemental File 1: normal distribution test for experimental data. [file 6668658.f5.docx]

**Supplemental File1 Normal distribution test for experimental data.**

Shapiro–Wilk test was used to analyze all the experimental data to ensure that most of the data in our study came from a normally distributed population. This test was analyzed by SPSS 20.0 software.

H0: the population distribution from which the sample comes obeys normal distribution (***P* > 0.05**).

H1: the population distribution of the sample does not obey the normal distribution (***P* ≤ 0.05**).

Under the test level of α = 0.05, the data could be considered to follow the normal distribution (***P* > 0.05**). The results are as follows:

| Real-time PCR analysis of *Eif2s3y* expression levels in different tissues of adult dairy goats in **Figure 1A**. | | | | | | |
| --- | --- | --- | --- | --- | --- | --- |
| Tissue Samples | Expression Levels | | | Shapiro-Wilk | | |
|  | 1 | 2 | 3 | W | df | *P* |
| Brain | 0.60 | 0.64 | 0.70 | 0.987 | 3 | 0.780 |
| Kidney | 0.78 | 0.84 | 0.89 | 0.997 | 3 | 0.900 |
| Heart | 1.02 | 1.17 | 1.43 | 0.977 | 3 | 0.706 |
| Liver | 1.45 | 1.81 | 1.99 | 0.964 | 3 | 0.637 |
| Ovary | 1.87 | 2.23 | 2.08 | 0.991 | 3 | 0.817 |
| Spleen | 1.87 | 2.01 | 2.21 | 0.990 | 3 | 0.806 |
| Lung | 2.85 | 3.02 | 2.13 | 0.887 | 3 | 0.345 |
| Testis | 5.48 | 5.25 | 4.95 | 0.994 | 3 | 0.855 |

| Real-time PCR analysis of *Eif2s3y* expression levels in the testes of dairy goats of different ages in **Figure 1B**. | | | | | | |
| --- | --- | --- | --- | --- | --- | --- |
| Month age | Expression Levels | | | Shapiro-Wilk | | |
|  | 1 | 2 | 3 | W | df | *P* |
| 1-month-old | 1.10 | 0.87 | 0.91 | 0.876 | 3 | 0.312 |
| 3-month-old | 3.40 | 3.88 | 3.23 | 0.930 | 3 | 0.487 |
| 6-month-old | 7.51 | 8.66 | 6.52 | 0.998 | 3 | 0.918 |
| 9-month-old | 11.21 | 10.13 | 12.52 | 0.997 | 3 | 0.894 |
| 12-month-old | 8.32 | 8.70 | 9.86 | 0.921 | 3 | 0.457 |
| 18-month-old | 13.69 | 14.57 | 15.54 | 0.999 | 3 | 0.946 |
| 24-month-old | 11.64 | 7.47 | 8.90 | 0.968 | 3 | 0.657 |
|  | | | | | | |
| RT-PCR analysis of the expression levels of *Eif2s3y*, *Pcna*, *Cyclin D*, *Zbtb16* in dairy goat SSCs transfected with Control-Vector or oeEif2s3y-Vector in **Figure 3G**. | | | | | | |
| Genes | Expression Levels | | | Shapiro-Wilk | | |
|  | 1 | 2 | 3 | W | df | *P* |
| *Control-Eif2s3y* | 0.96 | 0.99 | 1.05 | 0.964 | 3 | 0.637 |
| *Control-Pcna* | 0.87 | 1.03 | 1.04 | 0.794 | 3 | 0.100 |
| *Control-Cyclin D* | 1.13 | 0.99 | 0.93 | 0.949 | 3 | 0.567 |
| *Control-Zbtb16* | 1.16 | 0.91 | 0.96 | 0.893 | 3 | 0.363 |
| *oeEif2s3y-Eif2s3y* | 24.75 | 25.06 | 26.94 | 0.854 | 3 | 0.251 |
| *oeEif2s3y-Pcna* | 1.65 | 2.03 | 1.51 | 0.934 | 3 | 0.503 |
| *oeEif2s3y-Cyclin D* | 4.24 | 3.45 | 4.56 | 0.944 | 3 | 0.542 |
| *oeEif2s3y-Zbtb16* | 2.49 | 2.12 | 2.04 | 0.878 | 3 | 0.320 |

Gray intensity analysis of WB results normalized to GAPDH in **Figure 3I**.

| Proteins | Gray Intensity Analysis | | | | Shapiro-Wilk | | |
| --- | --- | --- | --- | --- | --- | --- | --- |
|  | 1 | 2 | 3 | 4 | W | df | *P* |
| Control-ZBTB16 | 1.05 | 0.97 | 0.91 | 0.92 | 0.926 | 4 | 0.572 |
| Control-eIF2γ | 1.04 | 0.95 | 1.05 | 0.96 | 0.821 | 4 | 0.145 |
| Control-PCNA | 1.03 | 0.99 | 0.93 | 1.15 | 0.791 | 4 | 0.086 |
| Control-Cyclin D | 1.10 | 1.03 | 1.00 | 0.87 | 0.956 | 4 | 0.756 |
| oeEif2s3y-ZBTB16 | 2.11 | 2.71 | 3.08 | 2.68 | 0.938 | 4 | 0.642 |
| oeEif2s3y-eIF2γ | 2.76 | 2.91 | 2.90 | 2.77 | 0.790 | 4 | 0.085 |
| oeEif2s3y-PCNA | 1.54 | 1.56 | 1.85 | 1.71 | 0.884 | 4 | 0.354 |
| oeEif2s3y-Cyclin D | 1.52 | 1.57 | 1.61 | 2.06 | 0.765 | 4 | 0.053 |

| RT-PCR analysis of the expression levels of *Eif2s3y*, *Pcna*, *Cyclin D*, *Zbtb16* in *shControl* and *shEif2s3y* SSCs in **Figure 4G**. | | | | | | |
| --- | --- | --- | --- | --- | --- | --- |
| Genes | Expression Levels | | | Shapiro-Wilk | | |
|  | 1 | 2 | 3 | W | df | *P* |
| *shControl-U6-1-Eif2s3y* | 0.96 | 0.99 | 1.05 | 0.964 | 3 | 0.637 |
| *shControl-U6-2-Eif2s3y* | 1.04 | 0.88 | 0.92 | 0.923 | 3 | 0.463 |
| *shControl-Pcna* | 1.01 | 1.06 | 0.94 | 0.991 | 3 | 0.817 |
| *shControl-Cyclin D* | 1.21 | 0.91 | 0.90 | 0.774 | 3 | 0.054 |
| *shControl-Zbtb16* | 1.13 | 0.98 | 0.96 | 0.837 | 3 | 0.206 |
| *shEif2s3y-U6-1-Eif2s3y* | 0.35 | 0.44 | 0.33 | 0.881 | 3 | 0.328 |
| *shEif2s3y-U6-2-Eif2s3y* | 0.15 | 0.16 | 0.23 | 0.842 | 3 | 0.220 |
| *shEif2s3y-Pcna* | 0.28 | 0.29 | 0.35 | 0.855 | 3 | 0.253 |
| *shEif2s3y-Cyclin D* | 0.62 | 0.64 | 0.48 | 0.842 | 3 | 0.220 |
| *shEif2s3y-Zbtb16* | 0.69 | 0.88 | 0.77 | 0.992 | 3 | 0.826 |

Gray intensity analysis of WB results normalized to GAPDH in **Figure 4I**.

| Proteins | Gray Intensity Analysis | | | | Shapiro-Wilk | | |
| --- | --- | --- | --- | --- | --- | --- | --- |
|  | 1 | 2 | 3 | 4 | W | df | *P* |
| shControl-ZBTB16 | 1.05 | 0.97 | 0.95 | 0.98 | 0.873 | 4 | 0.310 |
| shControl-eIF2γ | 1.13 | 1.17 | 1.05 | 0.87 | 0.773 | 4 | 0.062 |
| shControl-PCNA | 1.03 | 0.99 | 0.93 | 1.15 | 0.791 | 4 | 0.086 |
| shControl-Cyclin D | 1.04 | 0.95 | 1.05 | 0.96 | 0.821 | 4 | 0.145 |
| shEif2s3y-ZBTB16 | 0.30 | 0.34 | 0.40 | 0.39 | 0.917 | 4 | 0.519 |
| shEif2s3y-eIF2γ | 0.15 | 0.21 | 0.23 | 0.14 | 0.878 | 4 | 0.332 |
| shEif2s3y-PCNA | 0.22 | 0.36 | 0.25 | 0.37 | 0.847 | 4 | 0.216 |
| shEif2s3y-Cyclin D | 0.24 | 0.29 | 0.26 | 0.13 | 0.879 | 4 | 0.335 |

The testicular and epididymis weight/body mass ratio in two groups in **Figure 5C**.

| Groups | Shapiro-Wilk | | |
| --- | --- | --- | --- |
|  | W | df | *P* |
| Testis-Control | 0.881 | 20 | 0.314 |
| Testis-oeEif2s3y | 0.990 | 20 | 0.980 |
| Epididymis-Control | 0.833 | 20 | 0.146 |
| Epididymis-oeEif2s3y | 0.961 | 20 | 0.814 |

Statistical plots of the diameter of seminiferous tubules and the thickness of seminiferous epithelium from oeEif2s3y- and Control SSCs-transplanted mice in **Figure 5E and 5G**.

| Groups | Shapiro-Wilk | | |
| --- | --- | --- | --- |
|  | W | df | *P* |
| Testis-Control-Diameter | 0.975 | 30 | 0.732 |
| Testis-oeEif2s3y-Diameter | 0.965 | 30 | 0.474 |
| Testis-Control-Thickness | 0.988 | 30 | 0.986 |
| Testis-oeEif2s3y-Thickness | 0.979 | 30 | 0.863 |
| Epididymis-Control-Diameter | 0.985 | 34 | 0.914 |
| Epididymis-oeEif2s3y-Diameter | 0.979 | 34 | 0.729 |

| RT-PCR analysis of the expression of *Eif2s3y*, *Pcna*, *Zbtb16*, *Cyclin D* in Testis-Control and Testis-oeEif2s3y in **Figure 5H**. | | | | | | |
| --- | --- | --- | --- | --- | --- | --- |
| Genes | Expression Levels | | | Shapiro-Wilk | | |
|  | 1 | 2 | 3 | W | df | *P* |
| *Testis-Control-Eif2s3y* | 1.01 | 1.05 | 0.95 | 0.987 | 3 | 0.780 |
| *Testis-Control-Pcna* | 1.03 | 1.25 | 0.99 | 0.862 | 3 | 0.274 |
| *Testis-Control-Zbtb16* | 1.10 | 1.15 | 0.90 | 0.893 | 3 | 0.363 |
| *Testis-Control-Cyclin D* | 0.90 | 1.35 | 1.12 | 1.000 | 3 | 0.975 |
| *Testis-oeEif2s3y-Eif2s3y* | 4.32 | 4.12 | 4.07 | 0.893 | 3 | 0.363 |
| *Testis-oeEif2s3y-Pcna* | 2.32 | 2.42 | 2.27 | 0.964 | 3 | 0.637 |
| *Testis-oeEif2s3y-Zbtb16* | 5.52 | 5.42 | 5.07 | 0.907 | 3 | 0.407 |
| *Testis-oeEif2s3y-Cyclin D* | 2.11 | 2.56 | 1.78 | 0.992 | 3 | 0.831 |

| The percentage of EdU positive cells to total cells in **Figure 6B and 6D**. | | | | | | |
| --- | --- | --- | --- | --- | --- | --- |
| Groups | Percentage of EdU % | | | Shapiro-Wilk | | |
|  | 1 | 2 | 3 | W | df | *P* |
| Control+DMSO | 50.5 | 48.1 | 49.7 | 0.987 | 3 | 0.780 |
| oeEif2s3y+DMSO | 62.4 | 65.3 | 60.2 | 0.999 | 3 | 0.956 |
| Control+PD0325901 | 31.1 | 30.9 | 29.6 | 0.999 | 3 | 0.948 |
| oeEif2s3y+PD0325901 | 32.0 | 35.7 | 34.5 | 0.964 | 3 | 0.637 |
| shControl+DMSO | 44.4 | 46.5 | 47.4 | 0.983 | 3 | 0.747 |
| shEif2s3y+DMSO | 36.6 | 30.6 | 36.8 | 0.818 | 3 | 0.157 |
| shControl+TPA | 64.5 | 67.2 | 69.5 | 0.996 | 3 | 0.878 |
| shEif2s3y+TPA | 45.9 | 40.3 | 42.3 | 0.967 | 3 | 0.649 |

Gray intensity analysis of WB results normalized to GAPDH in **Figure 6F**.

| Groups | Gray Intensity Analysis | | | Shapiro-Wilk | | |
| --- | --- | --- | --- | --- | --- | --- |
|  | 1 | 2 | 3 | W | df | *P* |
| oeEif2s3y+DMSO-eIF2γ | 1.8687 | 1.8383 | 1.8739 | 0.857 | 3 | 0.259 |
| oeEif2s3y +DMSO-PCNA | 1.1516 | 1.1550 | 1.1662 | 0.913 | 3 | 0.429 |
| oeEif2s3y+DMSO-Cyclin D | 4.3691 | 4.3979 | 4.4559 | 0.964 | 3 | 0.634 |
| oeEif2s3y+DMSO-p-ERK | 1.4162 | 1.4274 | 1.3967 | 0.976 | 3 | 0.704 |
| oeEif2s3y+DMSO-Total ERK | 1.0154 | 1.0260 | 1.0363 | 1.000 | 3 | 0.984 |
| Control+DMSO-eIF2γ | 0.9987 | 1.0023 | 0.9990 | 0.812 | 3 | 0.144 |
| Control+DMSO-PCNA | 1.0112 | 0.9992 | 0.9896 | 0.996 | 3 | 0.878 |
| Control+DMSO-Cyclin D | 1.0073 | 1.0073 | 0.9942 | 0.861 | 3 | 0.272 |
| Control+DMSO-p-ERK | 1.0090 | 0.9872 | 1.0013 | 0.972 | 3 | 0.679 |
| Control+DMSO-Total ERK | 0.9983 | 1.0134 | 1.0185 | 0.924 | 3 | 0.468 |
| oeEif2s3y+PD0325901-eIF2γ | 1.8995 | 1.8612 | 1.8960 | 0.818 | 3 | 0.158 |
| oeEif2s3y+PD0325901-PCNA | 0.7182 | 0.7281 | 0.7208 | 0.930 | 3 | 0.489 |
| oeEif2s3y+PD0325901-Cyclin D | 0.5504 | 0.5470 | 0.5542 | 0.999 | 3 | 0.939 |
| oeEif2s3y+PD0325901-p-ERK | 0.8127 | 0.8232 | 0.8190 | 0.987 | 3 | 0.780 |
| oeEif2s3y+PD0325901-Total ERK | 1.0508 | 1.0537 | 1.0294 | 0.838 | 3 | 0.209 |
| Control+PD0325901-eIF2γ | 1.8995 | 1.8612 | 1.8960 | 0.975 | 3 | 0.694 |
| Control+PD0325901-PCNA | 0.7182 | 0.7281 | 0.7208 | 0.843 | 3 | 0.223 |
| Control+PD0325901-Cyclin D | 0.5504 | 0.5470 | 0.5542 | 0.999 | 3 | 0.927 |
| Control+PD0325901-p-ERK | 0.8127 | 0.8232 | 0.8190 | 0.931 | 3 | 0.491 |
| Control+PD0325901-Total ERK | 1.0508 | 1.0537 | 1.0294 | 0.900 | 3 | 0.385 |
| oeEif2s3y+TPA-eIF2γ | 1.8288 | 1.8334 | 1.8194 | 0.962 | 3 | 0.627 |
| oeEif2s3y+TPA-PCNA | 1.2892 | 1.2902 | 1.3027 | 0.805 | 3 | 0.127 |
| oeEif2s3y+TPA-Cyclin D | 3.7913 | 3.7813 | 3.8572 | 0.847 | 3 | 0.232 |
| oeEif2s3y+TPA-p-ERK | 2.0730 | 2.1154 | 2.0796 | 0.863 | 3 | 0.277 |
| oeEif2s3y+TPA-Total ERK | 0.9339 | 0.9289 | 0.9003 | 0.859 | 3 | 0.264 |
| Control+TPA-eIF2γ | 0.8255 | 0.8156 | 0.8324 | 0.989 | 3 | 0.804 |
| Control +TPA-PCNA | 1.4651 | 1.4998 | 1.5048 | 0.843 | 3 | 0.221 |
| Control+TPA-Cyclin D | 5.0505 | 4.9856 | 5.1172 | 1.000 | 3 | 0.985 |
| Control+TPA-p-ERK | 2.7827 | 2.8537 | 2.8241 | 0.991 | 3 | 0.817 |
| Control+TPA-Total ERK | 0.9410 | 0.8914 | 0.9122 | 0.991 | 3 | 0.823 |

Gray intensity analysis of WB results normalized to GAPDH in **Figure 6H**.

| Groups | Gray Intensity Analysis | | | Shapiro-Wilk | | |
| --- | --- | --- | --- | --- | --- | --- |
|  | 1 | 2 | 3 | W | df | *P* |
| shEif2s3y+DMSO-eIF2γ | 0.4657 | 0.4977 | 0.5970 | 0.919 | 3 | 0.450 |
| shEif2s3y+DMSO-PCNA | 0.5382 | 0.7548 | 0.5533 | 0.802 | 3 | 0.119 |
| shEif2s3y+DMSO-Cyclin D | 0.7769 | 0.6500 | 0.5970 | 0.947 | 3 | 0.555 |
| shEif2s3y+DMSO-p-ERK | 0.5253 | 0.5115 | 0.4106 | 0.839 | 3 | 0.211 |
| shEif2s3y+DMSO-Total ERK | 1.2063 | 1.0200 | 1.0314 | 0.796 | 3 | 0.104 |
| shControl+DMSO-eIF2γ | 0.9518 | 1.1265 | 0.9127 | 0.882 | 3 | 0.330 |
| shControl+DMSO-PCNA | 1.1582 | 0.8703 | 0.9592 | 0.954 | 3 | 0.585 |
| shControl+DMSO-Cyclin D | 0.8697 | 0.9256 | 0.9780 | 1.000 | 3 | 0.964 |
| shControl+DMSO-p-ERK | 1.0149 | 1.0323 | 1.0264 | 0.967 | 3 | 0.649 |
| shControl+DMSO-Total ERK | 0.9991 | 1.1093 | 1.0035 | 0.780 | 3 | 0.067 |
| shEif2s3y+TPA-eIF2γ | 0.4545 | 0.3791 | 0.4190 | 0.999 | 3 | 0.936 |
| shEif2s3y+TPA-PCNA | 1.0735 | 1.4524 | 1.2826 | 0.996 | 3 | 0.886 |
| shEif2s3y+TPA-Cyclin D | 1.1815 | 1.1879 | 1.0821 | 0.795 | 3 | 0.103 |
| shEif2s3y+TPA-p-ERK | 1.1657 | 1.1191 | 0.8867 | 0.871 | 3 | 0.299 |
| shEif2s3y+TPA-Total ERK | 1.1274 | 1.1623 | 1.2479 | 0.944 | 3 | 0.545 |
| shControl+TPA-eIF2γ | 0.8422 | 0.7234 | 0.8432 | 0.813 | 3 | 0.014 |
| shControl+TPA-PCNA | 2.7741 | 3.7343 | 2.8112 | 0.779 | 3 | 0.065 |
| shControl+TPA-Cyclin D | 1.0814 | 1.0411 | 0.9580 | 0.961 | 3 | 0.623 |
| shControl+TPA-p-ERK | 2.0132 | 2.1809 | 2.0809 | 0.988 | 3 | 0.788 |
| shControl+TPA-Total ERK | 0.9755 | 0.8907 | 0.9887 | 0.849 | 3 | 0.238 |

| RT-PCR analysis of the expression of *Zbtb16*, *GFRa1*, *Stra8* in primary cell and pure spermatogonia in **Supplemental Figure 1B**. | | | | | | |
| --- | --- | --- | --- | --- | --- | --- |
| Genes | Expression Levels | | | Shapiro-Wilk | | |
|  | 1 | 2 | 3 | W | df | *P* |
| Primary Cell*-Zbtb16* | 1.14 | 1.16 | 0.76 | 0.787 | 3 | 0.085 |
| Primary Cell*-GFRa1* | 1.07 | 0.88 | 0.95 | 0.977 | 3 | 0.712 |
| Primary Cell*-Stra8* | 1.18 | 0.84 | 0.95 | 0.960 | 3 | 0.616 |
| Pure Spermatogonia*-Zbtb16* | 5.28 | 4.32 | 4.19 | 0.838 | 3 | 0.209 |
| Pure Spermatogonia*-GFRa1* | 3.05 | 2.31 | 2.37 | 0.810 | 3 | 0.140 |
| Pure Spermatogonia*-Stra8* | 1.95 | 1.84 | 2.08 | 0.998 | 3 | 0.908 |
