# Supplementary material for: Eif2s3y Promotes the Proliferation of Spermatogonial Stem Cells by Activating ERK Signaling
Source: Stem Cells Int. 2021 Jan 29;2021:6668658. doi: 10.1155/2021/6668658 (PMC7869416; doi:10.1155/2021/6668658)
Supplement: Supplementary 6 — Supplemental File 2: experimental information for qRT-PCR. [file 6668658.f6.docx]

**Supplemental File.2 Experimental information for qRT-PCR.**

**Nucleic acid extraction:** Tissues and cells were harvested at the proper time, and total RNAs were extracted using Trizol reagent (RNAiso Plus, #9109, Takara Bio Inc, Japan). RNA integrity was analyzed by agarose gel electrophoresis and the concentration was determined using a NanoDrop 2000 Spectrophotometer (Thermo Scientific, USA). Aliquots of undiluted cDNA were stored in -20 °C and used for RT-PCR and real-time PCR. There were 1-5 million cells or 10-20 mg tissues each group used for total RNAs. The reagent without RNA-enzyme was used in the whole process.

| Samples | Concentration (ng/μL) | A260/280 |
| --- | --- | --- |
| adult dairy-Brain | 1265.32 | 1.96 |
| adult dairy-Kidney | 1461.83 | 1.87 |
| adult dairy-Heart | 1274.31 | 1.89 |
| adult dairy-Liver | 1238.67 | 1.87 |
| adult dairy-Overy | 994.52 | 1.84 |
| adult dairy-Spleen | 1467.16 | 1.89 |
| adult dairy-Lung | 1562.51 | 1.88 |
| adult dairy-Testis | 1581.29 | 1.81 |
| 1-month-old goat | 1574.88 | 1.81 |
| 3-month-old goat | 1589.44 | 1.89 |
| 6-month-old goat | 1888.16 | 1.81 |
| 9-month-old goat | 1664.88 | 1.82 |
| 12-month-old goat | 1616.32 | 1.87 |
| 18-month-old goat | 2355.74 | 1.90 |
| 24-month-old goat | 2692.31 | 1.93 |
| Control SSCs | 1193.53 | 2.13 |
| oeEif2s3y SSCs | 1406.81 | 2.05 |
| shControl SSCs | 1253.68 | 2.10 |
| shEif2s3y SSCs | 1194.41 | 2.12 |
| Testis-Control | 1270.17 | 1.92 |
| Testis-oeEif2s3y | 1345.10 | 1.92 |
| Primary cell | 1290.33 | 1.90 |
| Pure Spermatogonia | 1511.27 | 1.88 |

**• Reverse transcription:** Reverse transcription was carried out with 2 μg total RNA using RevertAid First Strand cDNA Synthesis Kit with gDNA Eraser (K1622, Lot 00887496, Thermo Fisher Scientific, Waltham, Massachusetts, USA). The usage is as follows: (https://www.thermofisher.com/order/catalog/product/K1622#/K1622)

Add the following reagents into a sterile, nuclease free tube on ice in the indicated order:

| Template RNA | Total RNA | 2 μg |
| --- | --- | --- |
| Primer | Oligo(dT) primer | 1 μL |
| Water, nuclease-free | | to 12 μL |
| Total volume | | 12 μL |
| 65 ℃ for 5 min | | |
| 5 × Reaction Buffer | | 4 μL |
| RiboLock RNase inhibitor (20 U/μL) | | 1 μL |
| 10 mM dNTP Mix | | 2 μL |
| RevertAid M-MuLVRT (200 U/μL) | | 1 μL |
| Total volume | | 20 μL |
| 42 ℃ for 60 min | | |
| 70 ℃ for 5 min | | |

The reverse transcription reaction product can be directly used in PCR applications or stored at -20°C for less than one week. For longer storage, -70°C is recommended

**• qPCR oligonucleotides:**

Supplemental Table 1 The sequence and length of primers used in qRT-PCR amplification.

| Gene name | Sense primer sequence (5ʹ−3ʹ) | Antisense primer sequence (5ʹ−3ʹ) | Product size /bp | Tm/℃ | GenBank |
| --- | --- | --- | --- | --- | --- |
| *Ei2s3y* | CCTTTGCTGCTTTCTTGTCTCC | TGCTGCTCCAGGTGGTCTTATT | 194 | 60 | >XM_018044897.1 |
| *Cyclin D* | GCGTACCCTGACACCAATCTC | CTCCTCTTCGCACTTCTGCTC | 183 | 60 | >XM_018043271.1 |
| *Pcna* | AGTGGAGAACTTGGAAATGGAA | GAGACAGTGGAGTGGCTTTTGT | 154 | 60 | >XM_005688167.3 |
| *Zbtb16* | CACCGCAACAGCCAGCACTAT | CAGCGTACAGCAGGTCATCCAG | 127 | 60 | >XM_018058857.1 |
| *GFRα1* | GGACAGGCAGCAGGAAATA | GTCTCCTGTCCCAGTCAAA | 201 | 60 | >XM_018041626.1 |
| *Stra8* | AAGGACAGCGGGGTTGAC | TCGGGAGTTTTTGAGTTGC | 170 | 60 | >XM_018047489.1 |
| *β-actin* | TGATATTGCTGCGCTCGT | CTTGAGGGTCAGGATGCC | 196 | 60 | >XM_018039831.1 |
| *GAPDH* | CGTGTCCGTTGTGGATCTGA | TGAAGTCGCAGGAGACAACC | 143 | 60 | >XM_005680968.3 |

**• qPCR target information:**

| Gene name | Sense primer target site (5ʹ−3ʹ) | Antisense primer target site (3ʹ−5ʹ) |
| --- | --- | --- |
| *Ei2s3y* | 1237 - 1216 | 1044 - 1065 |
| *Cyclin D* | 254 - 274 | 436 - 416 |
| *Pcna* | 723 - 744 | 876 - 855 |
| *Zbtb16* | 492 – 512 | 618 - 597 |
| *GFRα1* | 2856 - 2874 | 3056 -3038 |
| *Stra8* | 520 - 537 | 689 – 671 |
| *β-actin* | 134 – 151 | 329 – 312 |
| *GAPDH* | 816 – 835 | 958 – 939 |

**• qPCR protocol:**

RT-qPCR was conducted on a CFX Connect Real-Time System (Bio-Rad, California, USA) using SYBR premix Real Time PCR Kit (FP215-01, TianGen Biotech, Beijing, China) in accordance with the manufacturer’s instructions. To assess PCR efficiency, 10-fold serial dilutions of both *Gapdh* and *β-actin* plasmid cDNA were used to generate a standard curve for each assay plate. According to the standard curve, the PCR efficiency was determined to be 95% and 93% for *Gapdh* and *β-actin*, respectively. After the amplification, melt curves were obtained by slow heating from 60°C to 95°C at 0.1°C/s, with continuous fluorescence collection, confirming that only our specific product peaks were detected. Amplifications with different primers were performed with cDNA from the goat kidney, spleen, liver, heart, and muscle. As a control, a 143-bp fragment of the housekeeping *Gapdh* gene was amplified from the same cDNA preparations using *Gapdh* primers. The mRNA expression of *Eif2s3y* from SSCs was also detected.

Reaction system:

| Item | Volume |
| --- | --- |
| RT-PCR Mix (SYBR Green) | 10 μL |
| Sense primer | 0.5 μL |
| Antisense primer | 0.5 μL |
| cDNA | 1 μL |
| RNase free H_2_O | 8 μL |
| Total volume | 20 μL |

Complete thermocycling parameters：


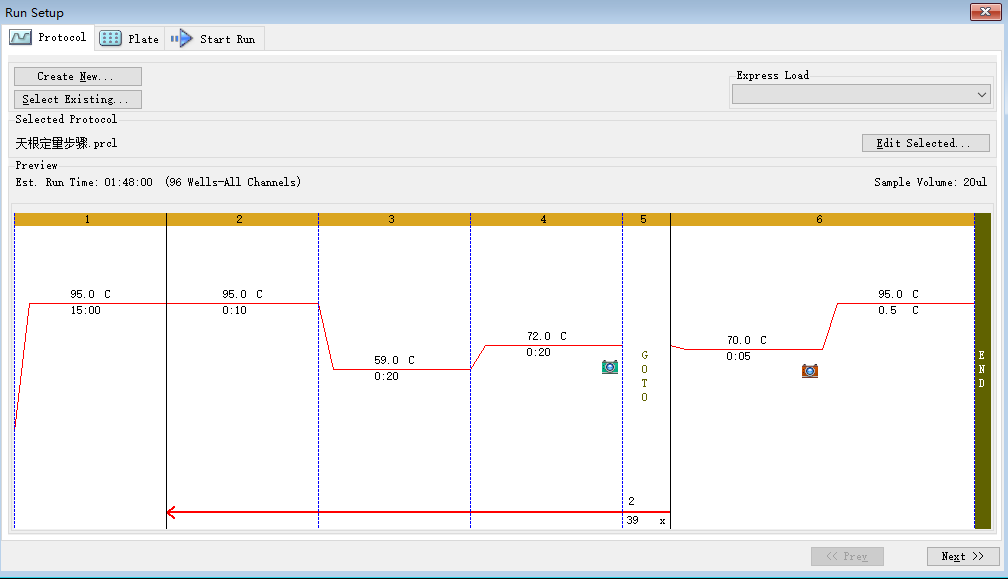


**• qPCR validation:**

RNA integrity was analyzed by agarose gel electrophoresis and the concentration was determined using a NanoDrop 2000 Spectrophotometer (Thermo Scientific, USA). Melting curves were constructed using the Dissociation Curves software to ensure that only a single product was amplified. All primer sequences were determined through established GenBank sequences. The PCR efficiency was evaluated and analyzed by agarose gel electrophoresis.

Cq of the no-template control (NTC) was N/A.

Melt peaks of *Eif2s3y* and *Gapdh*.


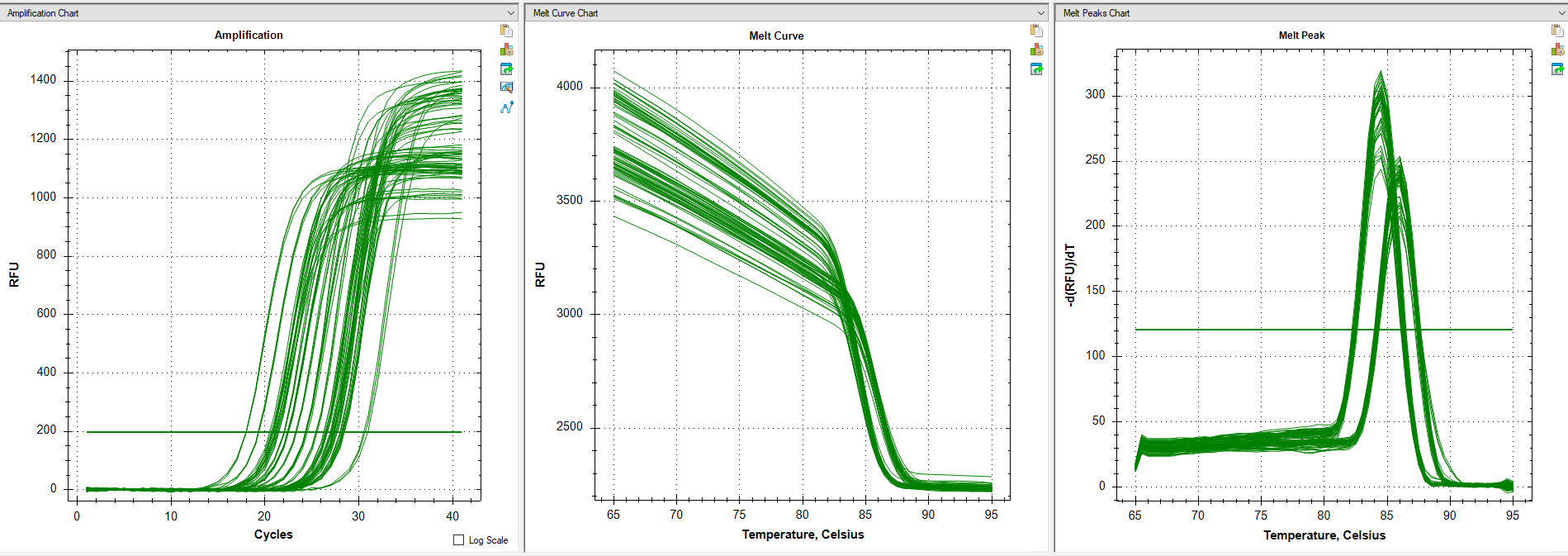


Melt peaks of other genes.


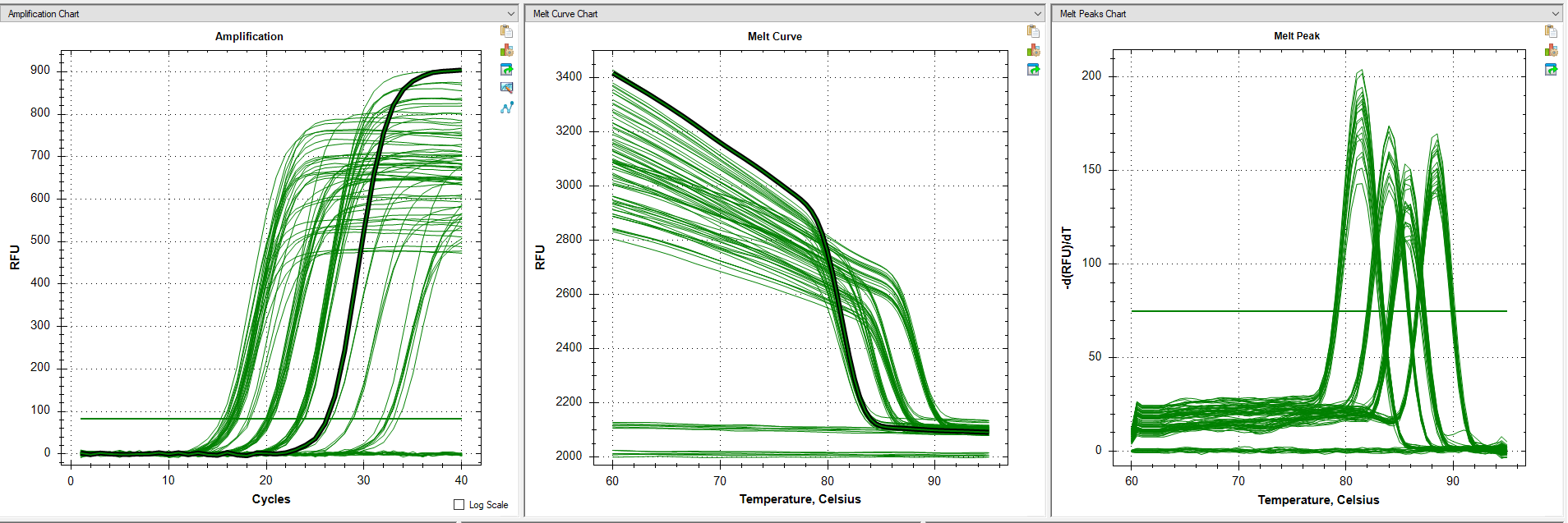


**• Data analysis:**

Relative gene expression was analyzed by the comparative Ct method (2^-ΔΔCt^ method). Reference genes were Gapdh and the mRNA expression of *Eif2s3y* was normalized against the expression of *Gapdh*. Data were expressed as the means ± SD and analyzed by SPSS version 20.0 (SPSS Inc., Chicago, IL, USA) and a two-tailed Student’s t test with Graphpad Prism software (La Jolla, CA). Three independent experiments were performed. Differences were considered significant at P<0.05.

Real-time PCR analysis of *Eif2s3y* expression levels in different tissues of adult dairy goats in Figure 1A.

| Organization | *Eif2s3y* | | | *P* value |
| --- | --- | --- | --- | --- |
| Brain | 0.60 | 0.64 | 0.70 | 0.000787795 |
| Kidney | 0.78 | 0.84 | 0.89 | 0.000806697 |
| Heart | 1.02 | 1.17 | 1.43 | 5.03499E-05 |
| Liver | 1.45 | 1.81 | 1.99 | 0.000162866 |
| Ovary | 1.87 | 2.23 | 2.08 | 0.000162866 |
| Spleen | 1.87 | 2.01 | 2.21 | 0.000183328 |
| Lung | 2.85 | 3.02 | 2.13 | 0.00318392 |
| Testis | 5.48 | 5.25 | 4.95 |  |

Real-time PCR analysis of *Eif2s3y* expression levels in the testes of dairy goats of different ages in Figure 1B.

| Month age | *Eif2s3y* | | | *P* value |
| --- | --- | --- | --- | --- |
| 1-month-old | 1.10 | 0.87 | 0.91 |  |
| 3-month-old | 3.40 | 3.88 | 3.23 | 0.002579418 |
| 6-month-old | 7.51 | 8.66 | 6.52 | 0.008010653 |
| 9-month-old | 11.21 | 10.13 | 12.52 | 0.004122707 |
| 12-month-old | 8.32 | 8.70 | 9.86 | 0.002783328 |
| 18-month-old | 13.69 | 14.57 | 15.54 | 0.001299502 |
| 24-month-old | 11.64 | 7.47 | 8.90 | 0.020379337 |

RT-PCR analysis of the expression levels of *Eif2s3y*, *Pcna*, *Cyclin D*, *Zbtb16* in dairy goat SSCs transfected with Control-Vector or oeEif2s3y-Vector in Figure 3G.

| Genes | Control | | | oeEif2s3y | | | *P* value |
| --- | --- | --- | --- | --- | --- | --- | --- |
| *Eif2s3y* | 0.96 | 0.99 | 1.05 | 24.75 | 25.06 | 26.94 | 0.000761443 |
| *Pcna* | 0.87 | 1.03 | 1.04 | 1.65 | 2.03 | 1.51 | 0.029263457 |
| *Cyclin D* | 1.13 | 0.99 | 0.93 | 4.24 | 3.45 | 4.56 | 0.009595987 |
| *Zbtb16* | 1.16 | 0.91 | 0.96 | 2.49 | 2.12 | 2.04 | 0.004114633 |

RT-PCR analysis of the expression levels of *Eif2s3y*, *Pcna*, *Cyclin D*, *Zbtb16* in *shControl* and *shEif2s3y* SSCs in Figure 4G.

| Genes | shControl | | | shEif2s3y | | | *P* value |
| --- | --- | --- | --- | --- | --- | --- | --- |
| *U6-1* | 0.96 | 0.99 | 1.05 | 0.35 | 0.44 | 0.33 | 0.000183497 |
| *U6-2* | 1.04 | 0.88 | 0.92 | 0.15 | 0.16 | 0.23 | 0.000742154 |
| *Pcna* | 1.01 | 1.06 | 0.94 | 0.28 | 0.29 | 0.35 | 0.000223676 |
| *Cyclin D* | 1.21 | 0.91 | 0.90 | 0.62 | 0.64 | 0.48 | 0.034374257 |
| *Zbtb16* | 1.13 | 0.98 | 0.96 | 0.69 | 0.88 | 0.77 | 0.034054862 |

RT-PCR analysis of the expression of *Eif2s3y*, *Pcna*, *Zbtb16*, *Cyclin D* in Testis-Control and Testis-oeEif2s3y in Figure 5H.

| Genes | Testis-Control | | | Testis-oeEif2s3y | | | *P* value |
| --- | --- | --- | --- | --- | --- | --- | --- |
| *Eif2s3y* | 1.01 | 1.05 | 0.95 | 4.32 | 4.12 | 4.07 | 0.000126848 |
| *Pcna* | 1.03 | 1.25 | 0.99 | 2.32 | 2.42 | 2.27 | 0.000743056 |
| *Zbtb16* | 1.10 | 1.15 | 0.90 | 5.52 | 5.42 | 5.07 | 7.59048E-05 |
| *Cyclin D* | 0.90 | 1.35 | 1.12 | 2.11 | 2.56 | 1.78 | 0.026047772 |

RT-PCR analysis of the expression of *Zbtb16*, *GFRa1*, *Stra8* in primary cell and pure spermatogonia in Supplemental Figure 1B.

| Genes | Primary Cell | | | Pure Spermatogonia | | | *P* value |
| --- | --- | --- | --- | --- | --- | --- | --- |
| *Zbtb16* | 1.14 | 1.16 | 0.76 | 5.28 | 4.32 | 4.19 | 0.004329172 |
| *GFRa1* | 1.07 | 0.88 | 0.95 | 3.05 | 2.31 | 2.37 | 0.01690943 |
| *Stra8* | 1.18 | 0.84 | 0.95 | 1.95 | 1.84 | 2.08 | 0.002191762 |
